# Interplay between $k$-core and community structure in complex networks

**DOI:** 10.1038/s41598-020-71426-8

**Published:** 2020-09-07

**Authors:** Irene Malvestio, Alessio Cardillo, Naoki Masuda

**Affiliations:** 1 Department of Engineering Mathematics, University of Bristol, Bristol, BS8 1UB UK; 2 Department of Computer Science and Mathematics, University Rovira i Virgili, 43007 Tarragona, Spain; 3 GOTHAM Lab – Institute for Biocomputation and Physics of Complex Systems (BIFI), University of Zaragoza, 50018 Zaragoza, Spain; 4 Department of Mathematics, University at Buffalo, Buffalo, NY 14260-2900 United States; 5 Computational and Data-Enabled Science and Engineering Program, University at Buffalo, State University of New York, Buffalo, NY 14260-5030 USA

**Keywords:** Statistical physics, thermodynamics and nonlinear dynamics, Complex networks

## Abstract

The organisation of a network in a maximal set of nodes having at least *k* neighbours within the set, known as $k$-core decomposition, has been used for studying various phenomena. It has been shown that nodes in the innermost $k$-shells play a crucial role in contagion processes, emergence of consensus, and resilience of the system. It is known that the $k$-core decomposition of many empirical networks cannot be explained by the degree of each node alone, or equivalently, random graph models that preserve the degree of each node (i.e., configuration model). Here we study the $k$-core decomposition of some empirical networks as well as that of some randomised counterparts, and examine the extent to which the $k$-shell structure of the networks can be accounted for by the community structure. We find that preserving the community structure in the randomisation process is crucial for generating networks whose $k$-core decomposition is close to the empirical one. We also highlight the existence, in some networks, of a concentration of the nodes in the innermost $k$-shells into a small number of communities.

## Introduction

Whenever a system can be abstracted as a set of units (*nodes*) interacting in pairs (*edges*), we can describe it as a network (also called a graph). Network analysis has proven to be a valuable framework to aid us to understand a plethora of phenomena taking place in many complex systems. Examples include cascades and collective behaviour in socio-technical systems, the emergence of cognitive functions in neural systems, the stability of chemical/biological systems, and the shape of spatially embedded systems, to cite a few^1–3^.

One of the advantages of the network representation is the possibility to probe the system in a coarse-grained manner, going beyond dyadic interactions by identifying high-order structures of the network^4,5^. Examples include tightly connected groups of nodes, i.e., communities^6^, multiscale coarse-grained structures^7^, core-periphery structure^8–10^, nested assembly of nodes^11^, rich clubs^12,13^, and the $k$-core^14,15^.

The $k$-core decomposition of a network is the maximal set of nodes that have at least *k* neighbours within the set^14,15^. The algorithm to extract the $k$-core consists in recursively removing the nodes having less than *k* connections. A $k$-shell is defined as the set of nodes belonging to the *k*^th^ core but not to the $(k + 1)$^th^ core^15^. The $k$-core decomposition has proven to be useful in a variety of domains such as identifying and ranking the most influential spreaders in networks, identifying keywords used for classifying documents, and in assessing the robustness of mutualistic ecosystem and protein networks^16,17^.

Models to generate random networks with specific features should help us to understand how the mechanisms governing the establishment of edges account for properties of empirical networks. Despite the vast range of applications of the $k$-core decomposition, to the best of our knowledge, there have been only few attempts to build models to generate networks with a given $k$-core structure. One indirect attempt to generate networks with a given $k$-core decomposition is the so-called brite model^18^. Originally, this model sought to replicate the features (including the $k$-core) of the Internet network at the Autonomous System (AS) level by mixing the mechanism of growth with preferential attachment^19,20^ and that of adding edges between already existing nodes. Another model aimed at generating networks with a $k$-core structure akin to an empirical one by leveraging the information stored in the so-called core fingerprint^21^. The core fingerprint corresponds to knowing the number of nodes in each $k$-shell, the number of intra-shell edges (i.e., those connecting nodes belonging to the same $k$-shell), and the number of inter-shell edges (i.e., those connecting nodes belonging to different $k$-shells) of a given network. Moreover, the authors qualitatively compared the Internet AS networks and synthetic networks preserving the core fingerprint of the original networks using several indicators^21^. More recently, models based on modified versions of the so-called configuration model have been proven to be effective in generating networks with $k$-core structure akin to that of empirical networks^22,23^. In a nutshell, in these models the edge stubs attached to each node are divided into two groups: red and blue. Red stubs can create any edges regardless of the $k$-shell structure. Blue stubs only form edges connecting nodes belonging to distinct $k$-shells. Among the possible pairs of stubs’ colours, only the blue-blue pair is forbidden.

As mentioned above, another type of mesoscale structure is communities. Although there is not a univocal definition of what a community is, in general the community refers to a group of nodes that are more tightly connected between each other than with the other nodes of the network^6^. Communities are also defined by the concept of stochastic equivalence, i.e., nodes in the same group/community interact, on average, with nodes in other groups in the same way^24^. Methods based on different definitions of communities may return different partitions of the node set. However, there is often some consistency between those partitions, which indicates the presence of groups of nodes acting like the building blocks of communities^25^. The presence of communities is an important large-scale characteristic of many empirical networks because a system’s different functions tend to be located in different communities (e.g., in functional brain networks^26^ and protein-protein interaction networks^27^). Moreover, it has been proven that communities play a role in the resilience of the system^28^ and the presence of triangles^29^, as well as in the emergence of collective behaviour including synchronisation^30^, the emergence of cooperation^31,32^, spreading of a pandemic^33^, and the attainment of consensus^34,35^.

Although $k$-core and communities are two ways of decomposing the same network, there may be overlaps or intricate relationships between them. In the present paper, we study the relation between the $k$-core decomposition and the community structures of several empirical and synthetic networks. In particular, we leverage the work of Alvarez-Hamelin et al.^36^ and confirm that the nodes’ degrees (i.e., their number of edges) alone are not capable of reproducing the network’s $k$-shell structure. We find that one has to include information about the community structure to obtain networks whose $k$-core decomposition looks sufficiently close to the empirical one. We also highlight the existence of a concentration-like phenomenon of the innermost $k$-shells into a small number of communities, which is stronger in some data sets than others.

## Results

### Degree-based reconstruction of the $k$-core

As stated above, various studies on networks leverage the $k$-core decomposition to extract insightful information from networks. However, less studies have asked which mechanisms are sufficient for explaining generation of networks having empirically observed patterns of $k$-core decomposition. More specifically, Alvarez-Hamelin et al. found that networks generated using the configuration model^37^ having a Poisson or power-law degree distribution do not display a $k$-core structure similar to the one displayed by the AS network^36^. Using the results of Alvarez-Hamelin et al. as a starting point, given an empirical network *G* with *N* nodes, we check whether its $k$-core decomposition can be reproduced solely from the degree of each node *i* (i.e., the number of edges that node *i* has), denoted by $k_i$. We generated random networks by a standard configuration model preserving the degree of each node of *G*, which we denote by deg (see “Methods” section for details).

**Figure 1:**
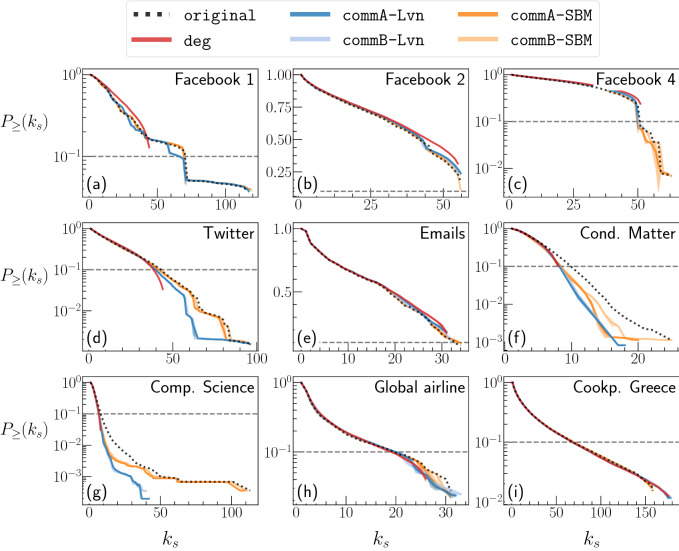
Survival function of the probability distributions of the $k$-shell index, i.e., $P_{\ge }(k_\text {s})$ as a function of $k_\text {s}$ for the original network (dotted line) and shuffled networks (solid line). Each panel corresponds to a data set, i.e., Facebook 1 (panel **a**), Facebook 2 (**b**), Facebook 4 (**c**), Twitter (**d**), Emails (**e**), Cond. Matter (**f**), Comp. Science (**g**), Global airline (**h**), and Cookpad Greece (**i**). The horizontal dashed lines indicate that $P_{\ge }(k_\text {s}) = 0.1$. Results are averaged over 10 different runs of each shuffling method, and the shaded areas (when visible) represent the standard deviations.

We have analysed several empirical networks encompassing social, technological, linguistic, and transportation systems whose main properties are summarised in Table 1. In Fig. 1, we show the survival function of the probability distributions of the $k$-shell index, $P_{\ge }(k_\text {s})$ (i.e., fraction of nodes whose $k$-shell index is larger than or equal to $k_\text {s}$), for a selection of data sets, compared across the original networks and their synthetic counterparts (see Supplementary Fig. S1 in SM for the other data sets). Figure 1 indicates that the degree of each node is not sufficient for reproducing the $k$-core structure of the original networks because $P_{\ge }(k_\text {s})$ for deg considerably deviates from that for the original networks. This result is consistent with the previous results^36^. In fact, we find that fixing the degree of each node is sufficient to recover the $k$-core profile in some networks. For these networks the empirical and deg networks are not too different in terms of $P_{\ge }(k_\text {s})$ (e.g., Facebook 2 and Cookpad networks). We point out two main differences in $P_{\ge }(k_\text {s})$ between the empirical and deg networks. First, for most data sets, the largest $k_\text {s}$ value, which is denoted by *D* and called the degeneracy, is considerably smaller for the networks generated by deg than the original networks. Second, the $P_{\ge }(k_\text {s})$ of some empirical networks have plateaus and abrupt drops in $k_\text {s}\le D$. The plateaus imply that some of the $k$-shells are completely or almost empty, whereas the abrupt drops indicate that some $k$-shells are more densely populated than those adjacent to them. In contrast, $P_{\ge }(k_\text {s})$ for the deg networks does not have a notable plateau or drop in $k_\text {s}\le D$. Therefore, in the deg networks, all the $k$-shells up to $k_\text {s}= D$ are populated, and there is no $k$-shell that is substantially more populated than its adjacent $k$-shells.

**Table 1:** Main properties of the data sets used in the present study. *N*: number of nodes, *L*: number of edges, $\langle k \rangle$: average degree, $k_{\max }$: maximum degree, $\langle ks \rangle$: average value of the $k$-shell index, *D*: maximum value of the $k$-shell index, $N_\text {c}^{{\texttt {Lvn}{}}}$, $Q^{\texttt {Lvn}{}}$: number of communities determined by the Louvain method and the corresponding modularity, respectively, $N_\text {c}^{{\texttt {SBM}{}}}$, $Q^{\texttt {SBM}{}}$: number of communities determined by the SBM and the corresponding modularity, respectively.

Data set	*N*	*L*	$\langle k \rangle$	$k_{\max }$	$\langle k_\text {s}\rangle$	*D*	$N_\text {c}^{{\texttt {Lvn}{}}}$	$Q^{\texttt {Lvn}{}}$	$N_\text {c}^{{\texttt {SBM}{}}}$	$Q^{\texttt {SBM}{}}$	References
Facebook 1	4039	88234	43.691	1045	26.880	115	16	0.835	62	0.551	^55,74^
Facebook 2	6386	217662	68.168	930	35.712	56	19	0.419	198	0.158	^56–58,75^
Facebook 3	2235	90954	81.391	467	44.508	63	8	0.436	87	0.139	^56–58,76^
Facebook 4	11247	351358	62.480	415	32.413	63	10	0.438	274	0.193	^56–58,77^
Facebook 5	27737	1034802	74.615	2555	38.681	81	18	0.470	547	0.172	^56–58,78^
Twitter	81306	1342296	33.018	3383	17.762	96	73	0.808	510	0.511	^55,79^
Web-blogs	1490	16715	22.436	351	12.154	36	275	0.426	17	0.076	^60,80^
Emails	1005	16064	31.968	345	17.063	34	26	0.410	33	0.232	^81–83^
Cond. Matter	23133	93439	8.078	279	4.900	25	619	0.730	203	0.633	^83,84^
Comp. Science	317080	1049866	6.622	343	4.215	113	209	0.822	676	0.726	^59,85,86^
Global airline	3376	19179	11.362	248	6.123	31	26	0.665	40	0.311	^61^
Words	146005	656999	9.000	1008	5.289	31	378	0.759	548	0.583	^59,87,88^
Cookpad Greece	32235	745178	46.234	8196	23.709	158	40	0.166	76	0.020	–
Cookpad Spain	122158	1749751	28.647	12637	14.547	162	262	0.270	90	0.035	–
Cookpad UK	13758	47525	6.909	1880	3.558	33	199	0.350	8	0.114	–

A more quantitative comparison of distribution $P_{\ge }(k_\text {s})$ between the empirical and deg networks may be done by, for example, the Kolmogorov-Smirnov (KS) test^38^. However, because a majority of the nodes usually belongs to outer $k$-shells, (i.e., set of nodes with small $k_\text {s}$ values) and Fig. 1 shows that the strongest discrepancies between the two distributions tend to occur at large $k_\text {s}$ values, the KS test fails to grasp the differences at large $k_\text {s}$ values that we are mostly interested in. Therefore, we compare the $k$-core decomposition of the empirical and deg networks using four indicators, i.e., the relative difference in the average $k$-shell index, $\Delta \langle k_\text {s} \rangle$, the relative difference in the network’s degeneracy, $\Delta D$, the Jaccard score, *J*, and Kendall’s, $\tau _K$ of the nodes belonging to the top 10% (i.e., innermost $k$-shells) of the $P_{\ge }(k_\text {s})$ distribution. The average of each indicator over all the data sets for the networks obtained with the deg shuffling method is equal to $\langle \Delta \langle k_\text {s} \rangle \rangle = 0.052 \pm 0.056$, $\langle \Delta D \rangle = 0.302 \pm 0.288$, $\langle J \rangle = 0.563 \pm 0.194$, and $\langle \tau _K \rangle = 0.763 \pm 0.176$. The value of $\langle \Delta \langle k_\text {s} \rangle \rangle$ indicates that $\langle k_\text {s} \rangle$ is only $\approx 5\%$ different between the original and deg networks on average. However, their degeneracy differs by $\approx 30\%$ on average. The $\langle J \rangle$ and $\langle \tau _K \rangle$ values inform us that innermost $k$-shells of the original networks and those of the deg networks tend to share approximately half of the nodes, albeit their ranking seems to be fairly preserved. Supplementary Table S1 reports the values of each indicator.

### Community-aware reconstruction of the $k$-core

We have seen that the degree distribution by itself does not reproduce main features of the $k$-shell index distribution. An alternative feature that may explain the $k$-shell index distribution is the community structure. For this reason, we generated synthetic networks that preserve both the degree of each node and the community structure, ${\mathcal {C}} = \{ C_1, \ldots , C_{N_\text {c}} \}$, where $N_\text {c}$ is the number of communities of the original network. To account for the multiple definitions of what a community is, we identified the communities of each network using two methods: the Louvain method^39^, denoted by Lvn, and the degree-corrected stochastic block model^40^, denoted by SBM. In combination with each of the two community detection methods, we considered two rewiring methods preserving ${\mathcal {C}}$ and the degree of each node, denoted by commA and commB. Method commA preserves the exact number of inter- and intra-community edges at the level of single communities. Method commB preserves the number of inter- and intra-community edges for each node.

Figure 1 indicates that preserving the community structure in addition to the degree of each node improves the similarity in $P_{\ge }(k_\text {s})$ between the empirical and synthetic networks, especially at large $k_\text {s}$ values, which correspond to inner $k$-shells. In particular, commA and commB generate networks whose *D* value tends to be closer to the empirical value than deg does. Furthermore, $P_{\ge }(k_\text {s})$ for commA and commB tends to have plateaus and abrupt drops at $k_\text {s}\le D$ similarly to the empirical networks. Overall, synthetic networks preserving the SBM community structure have a $k$-core decomposition more akin to the empirical one than those preserving the Lvn community structure. This observation is quantitatively supported by the values of the four indices reported in Supplementary Table S1.

**Figure 2:**
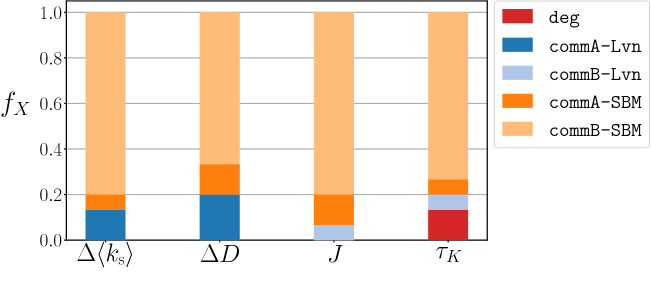
Performances of different shuffling methods in terms of four indicators. We report the fraction of data sets for which a given combination of the shuffling method and the community detection method yields an indicator’s value closest to that for the original network. Each bar refers to an indicator, i.e., average $k$-shell’s difference, $\Delta \langle k_\text {s} \rangle$, degeneracy’s difference, $\Delta D$, Jaccard score, *J*, and Kendall’s tau, $\tau _K$.

To obtain an overview of the performances of different network randomisation methods, in Fig. 2 we show the fraction of data sets, $f_X$, for which a certain shuffling method generates a $k$-core decomposition that is the most similar to that of the empirical network according to each indicator. The figure indicates that commB-SBM (i.e., the commB shuffling method that preserves the community structure determined by SBM) performs the best in mimicking the $k$-shell index features for approximately 65–80% of the data sets, depending on the indicator. Detailed results for the performance of each method for each empirical network are shown in Supplementary Fig. S2 and Supplementary Table S1.

One issue of Lvn is that it cannot discover small communities^6,41^. One way to mitigate this limitation is to introduce in Lvn a resolution parameter, $r \in (0,1]$, regulating the resolution scale. It is possible to detect small communities when *r* is small, whereas the original Lvn corresponds to $r=1$^42^. We denote by LvnR the Louvain method with $r<1$, i.e., with a resolution higher than that used by Lvn. In Sect. 2 of SM we report whether preserving the communities found using LvnR instead of Lvn improves our ability to reproduce the $k$-core decomposition of the original network. We found that LvnR performs better than Lvn (Supplementary Figs. S3 and S4) but worse than SBM in general (Supplementary Fig. S5).

Imposing the simultaneous conservation of each node’s degree and community structure may result in synthetic networks that are not substantially different from the original ones. To exclude this possibility, we computed the Jaccard score, $J({\mathcal {L}},{\mathcal {L}}^\prime )$, (see Eq. (3)) for the sets of edges, ${\mathcal {L}}$ and ${\mathcal {L}}^\prime$, of the original and shuffled networks, respectively. The values of *J* approximately fall between 0.01 and 0.5, confirming that the set of edges – hence, the networks – are considerably different.

The results presented so far suggest that preserving the community structure improves the preservation of the $k$-core decomposition of the original network. Therefore, the mere presence of a community structure may be enough to preserve the main features of the $k$-core decomposition of the original networks. To test this possibility, we applied the $k$-core decomposition to networks with communities generated using the LFR model^43^ (see Sect. 3 of SM). The plots of $P_{\ge }(k_\text {s})$ shown in Supplementary Figs. S7–S10 indicate that the presence of a community structure alone is not sufficient for producing major features of the $k$-core structure of the empirical networks. Specifically, the $P_{\ge }(k_\text {s})$ of the networks generated by the LFR model is always smooth and shows neither plateaus nor abrupt drops as $k_\text {s}$ increases. Moreover, with the LFR, $k_\text {s}$ is narrowly distributed, i.e., $\max (k_\text {s}) - \min (k_\text {s}) \approx 10$. These differences between the $k$-core structure of the LFR model and that of empirical networks are not sensitive to the value of the mixing parameter, $\mu$, of the LFR model, which controls how distinct the communities are. It should also be noted that for the LFR model, as for the empirical network, the commB-SBM generates networks that are the most similar to the original LFR networks among the different shuffling methods in terms of $P_{\ge }(k_\text {s})$.

### Overlap between communities and $k$-core

**Figure 3:**
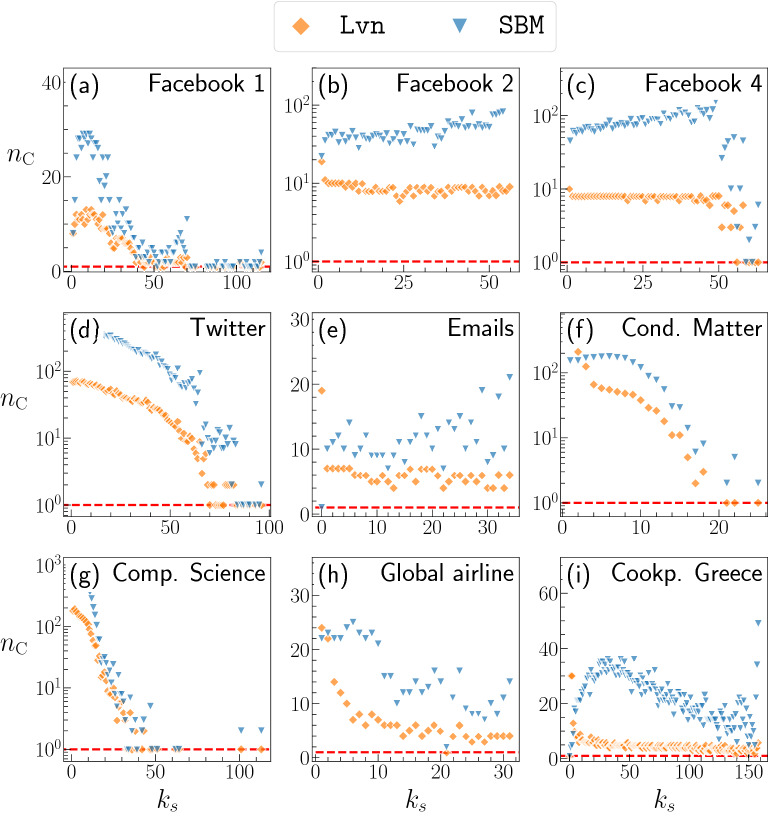
Number of different communities, $n_\text {C}(k_\text {s})$, that the set of nodes of a given $k$-shell value, $k_\text {s}$, overlaps. The horizontal dashed line is a guide to the eyes showing $n_\text {C}(k_\text {s}) = 1$. Each panel accounts for a different data set (see the caption of Fig. 1 for the details). For each data set, we show the results corresponding to the community structure obtained using either Lvn or SBM.

Preserving the community structure in addition to the node’s degree can lead to preservation of features of the $k$-core structure possibly because nodes with high values of $k_\text {s}$ form a $k$-core which tend to belong to the same community. To examine this possibility, we show the number of communities to which the nodes of a given $k$-shell belong, $n_\text {C}(k_\text {s})$, in Fig. 3 (see Supplementary Fig. S11 for the other data sets). Although each data set shows a distinct pattern, for many data sets, inner $k$-shells (i.e., nodes with large $k_\text {s}$ values) are concentrated into one or a few communities. The concentration effect is particularly noticeable for some data sets, e.g., Facebook 1 and Twitter. To check whether the number of communities per $k$-shell is merely a byproduct of the random combinatorial effect owing to the number of communities, the distribution of the community size, and the distribution of $k_\text {s}$, we computed a random assignments of the nodes to communities and then calculated $n_\text {C}(k_\text {s})$ for each $k_\text {s}$ value (see Sect. 4 and Supplementary Fig. S12 of the SM). We have found that the nodes in each $k$-shell are almost always more concentrated into a smaller number of communities than what is expected by the random assignment of the nodes to communities for all the data sets and community detection methods, with the only exception of SBM for Cookpad’s data sets. This finding is in agreement with the previous result that nodes with high $k_\text {s}$ tend to belong to the same community, which has been observed in networks embedded into hyperbolic spaces^44,45^. In particular, we observe a strong concentration of the $k$-shells into a few communities for the Facebook 1, Twitter, Cond. Matter, Comp. Science, and Words networks, which are those showing a more pronounced difference in the values of *D* between the original and deg networks.

## Discussion

The information encoded in the degree of each node is not sufficient for generating networks with a $k$-core structure that is similar to those of empirical networks^36^. This gap of knowledge calls for the design of generative models of networks beyond the configuration model. Such models are expected to be useful to generate benchmark networks and to understand the mechanisms behind the emergence of the $k$-core. To the best of our knowledge, few models are available to generate networks with a given $k$-core decomposition^21–23^.

In the present study, we investigated how much the combination of the nodes’ degrees and community structure accounts for $k$-core structure of empirical networks. Given a network *G*, we randomly shuffled *G*’s edges to generate its synthetic counterparts preserving each node’s degree and/or community structure of *G*. We found that randomised networks preserving the community structure obtained through a stochastic block model showed a $k$-shell index distribution that was reasonably similar to the distribution for the original networks. The success of the stochastic block model in mimicking the features of the $k$-core decomposition might be due to its ability to approximate the mesoscale structures of networks with a good accuracy^24,46^, including communities. We also sought to understand more the relationship between $k$-core and communities by studying networks generated by the LFR model which enables us to control the extent to which the communities are distinguished from each other. However, regardless of whether or not different communities are relatively distinguished from each other in a network, the $k$-shell index distribution of LFR networks does not show the same features as those observed in the empirical networks. Finally, we have investigated the overlap between communities and $k$-shells and found that, in some empirical networks, the nodes in inner $k$-shells are concentrated into a small number of communities much more so than a randomised counterpart. This result is in agreement with the observations made for networks embedded in hyperbolic spaces^44,45^. Up to our numerical efforts, the concentration is observed if and only if the empirical network and its deg counterpart are substantially different in terms of their $k$-core decomposition. The concentration suggests that inner $k$-shells may perform specific functions in such networks, corresponding to the functions of the communities they belong to as observed in, for instance, functional brain networks^26^ and protein-protein interaction networks^27^.

The “community aware” rewiring mechanisms introduced in this paper can be used for assessing whether or not a given property of a network is a direct expression of its community structure. One example of such an approach is given in^47^, where the authors have improved the robustness against attacks on a network while keeping its community structure. In that case, the method only preserves the communities and alters the connectivity pattern by increasing the density of intra-community edges as well as changing the edges between communities. It may be interesting, instead, to check whether the robustness of the network can be improved even when one also preserves the degree of the nodes using our community-aware rewiring mechanisms.

One viable extension of our work is to the case of *k*-peak graph decomposition method^48^. In Ref.^48^, the authors argue that for networks with communities, the $k$-core decomposition should be performed locally rather than globally, thus returning the *k*-peak decomposition of each of the system’s regions. The rationale behind this approach is to avoid that, if the network contains regions with different densities of edges, the standard $k$-core decomposition would fail to recognise local core nodes in sparser regions. Studying the evolution of the *k*-peak decomposition in response to the rewiring of the connections may unveil salient features of complex systems. Another possible direction of research is to concatenate the information encoded into the $k$-shell index, $k_\text {s}$, with the one provided by the so-called onion decomposition (OD)^49^. The OD is an extension of the $k$-core decomposition where a node is labelled with both its $k_\text {s}$ and its layer index. The layer of a node *i* represents the iteration number with which node *i* is removed in the recursive pruning process of the $k$-core decomposition. The OD provides a further characterisation of the structure of the network than the $k$-core, revealing, for example, how tree-like the network is.

Summing up, in this work we have analysed the interplay between the $k$-core decomposition and community structure of networks. Understanding such a relationship is useful not only owing to the broad range of applications of $k$-core decomposition, but also to inform the design of models capable of generating networks with both a community structure and $k$-core’s features beyond those explainable by the degree distribution. Such models may stand on, for instance, the stochastic block model^40^, the enhanced configuration model based on maximum entropy^50^, or the hierarchical extension of the LFR model^51^. Alternatively, models based on microscopic growth mechanisms such as triadic closure^52,53^ or modified preferential attachment^54^ may deserve further investigation.

## Methods

### Data

We have considered networks corresponding to systems of different types: from social to technological, from semantic to transportation. Table 1 summarises main properties of such networks. Except for Cookpad networks, all the data sets are publicly available and have been retrieved from the Stanford Large Network data set Collection^55^ (Facebook 1, Twitter, Emails, and Cond. Matter), the Network Repository^56–58^ (Facebook 2, 3, 4, and 5), the Koblenz Network Collection (KONECT)^59^ (Comp. Science, and Words), Mark E. J. Newman’s personal network data repository^60^ (Web-blogs), and the OpenFlights data repository^61^ (Global airline). In the following text, we provide a brief description of each data set.

- *Facebook and Twitter* These networks describe social relationships. Nodes are people. Edges represent their friendship relations.
- *Web-blogs* This network is composed of the hyperlinks (edges) between weblogs on US politics (nodes) recorded in 2005.
- *Emails* This is a network of email data from a large European research institution. Nodes are people. Edges connect pairs of individuals who have exchanged at least one e-mail.
- *Cond. Matter and Comp. Science* The former network is the co-authorship network of the authors of preprint manuscripts submitted to the Condensed Matter Physics arXiv e-print archive from January 1993 to April 2003. The latter network is similarly defined using manuscripts appearing in the DBLP computer science bibliography, using a comprehensive list of research papers in computer science. The submission time of the papers of the DBLP collection is unavailable. A node is an author. An edge represents the existence of at least one manuscript co-authored by two authors.
- *Global airline* In this network nodes are airports across the globe. An edge indicates direct commercial flights between two airports.
- *Words* This network accounts for the lexical relationships among words extracted from the WordNet data set. Nodes are English words. Edges are relationships (synonymy, antonymy, meronymy, etc.) between pairs of words.
- *Cookpad* These networks are extracted from the Cookpad online recipe sharing platform^62^. Users can post and browse recipes, as well as interact with other users through recipes in multiple ways including liking, sharing, and posting a comment. The platform is present in many countries (e.g., Japan, Indonesia, United Kingdom, and Italy). Here, we consider the data collected from September to November of 2018 in Greece, Spain, and the United Kingdom, separately for each country. In the three networks, nodes are users. An edge between a pair of users exists if one or more of the following types of events takes place: like or follow a user, viewing, bookmarking, commenting, or making a cooksnap of another user’s recipe.

All the networks considered in this work are treated as undirected and unweighted, even when the original data contains more information. Finally, we also consider synthetic networks, generated using the LFR (Lancichinetti–Fortunato–Radicchi) model^43^ (see Sect. 3 of SM for details).

### Network shuffling

Given a network, *G*, with *N* nodes and *L* edges, we generate a randomised counterpart, $G^\prime$, that has the same nodes and the same number of edges by shuffling the edges of *G*. We consider three shuffling methods denoted by deg, commA, and commB; each shuffling method preserves different properties of *G*. The shuffling consists in selecting uniformly at random two edges (*a*, *b*) and (*c*, *d*), and replacing them with, e.g., (*a*, *c*) and (*b*, *d*), if the swapping of the edges is accepted. An attempt to swap edges is accepted, in which case we call the swapping effective, if and only if it respects the rule of the specific shuffling method and the swapping does not generate self-loops or multiple edges. We continued the shuffling until we carried out 2*L* effective swaps, such that an edge was swapped four times on average.

In the following text, we provide the details of each shuffling method. Assume that network *G* partitions into communities such that the set of the communities is ${\mathcal {C}} = \{C_1, \ldots , C_{N_\text {c}} \}$, where $N_\text {c}$ is the number of communities. Furthermore, let $g(i) \in {\mathcal {C}}, \; i = 1, \dots , N$, be the community to which the *i*th node belongs and $k_i$ be the degree of node *i*. We have:

- *Degree-preserving shuffling* (deg) This method preserves degree $k_i$ of each node *i* and is equivalent to the configuration model^37^.
- *Community-preserving shuffling of type A* (commA) On top of the degree of each node, this method preserves the total number of edges within each community and between each pair of communities. In attempts to swap edges, we replace two randomly selected edges (*a*, *b*) and (*c*, *d*) by (*a*, *c*) and (*b*, *d*) if and only if an end node of edge (*a*, *b*) and an end node of edge (*c*, *d*) belong to the same community (i.e., if $g(b) = g(c)$ or $g(a) = g(d)$).
- *Community-preserving shuffling of type B* (commB) Like commA, this method preserves the degree of each node and the number of edges within each community and between each pair of communities. In contrast with commA, the commB method preserves the numbers of edges within and across communities for each node, and not only for each community or pairs of communities. Given two selected edges (*a*, *b*) and (*c*, *d*), we replace them with (*a*, *c*) and (*b*, *d*) if and only if the two new edges connect the same community pairs as before the swapping (i.e., $g(b) = g(c)$ and $g(a) = g(d)$).

### Comparison of the $k$-core decomposition

To assess the similarity between the $k$-core decomposition of the original network, *G*, and of its shuffled counterpart, $G^\prime$, we used four indicators: the average $k$-shell index, $\langle k_\text {s} \rangle$, the network’s degeneracy, *D*, the Jaccard score, *J*, and the generalised Kendall’s tau, $\tau _K$. The indicator $\langle k_\text {s} \rangle$ explicitly depends on all the nodes in the network, whereas *D*, *J* and $\tau _K$ only depend on the nodes belonging to the innermost $k$-shell(s). We use the latter three indicators because, although a majority of nodes tends to belong to outer $k$-shells, it is a difference in the tails of the $k_\text {s}$ distributions that often affect functions of networks such as the impact of influencers in contagion processes^63^. The four indicators are defined as follows.The average of the $k$-shell index, $\langle k_\text {s} \rangle$, is equal to

1 $$\begin{aligned} \langle k_\text {s} \rangle = \frac{1}{N} \sum \limits _{i=1}^N k_\text {s}(i)\,, \end{aligned}$$

where $k_\text {s}(i)$ is the $k$-shell index of node *i*. The degeneracy, *D*, of a network *G* is given by^64^

2 $$\begin{aligned} D = \max _{i \in G} \{ k_\text {s}(i) \} \,. \end{aligned}$$

Rather than using these raw indicators, to compare across the different data sets, we compute their relative difference between the empirical network and its shuffled counterpart given by $\Delta X = \left|X_G - X_{G^\prime } \right|/ X_G$, where $X \in \{ \langle k_\text {s} \rangle , D \}$.

To compute *J* and $\tau _K$, we need to define a criterion to select nodes belonging to the innermost $k$-shells. We decided to confine the comparison to the nodes whose $k_\text {s}$ falls within the top $10\%$ among the *N* nodes. The horizontal lines in Fig. 1 indicate the threshold values of $k_\text {s}^\star$ such that $P_{\ge }(k_\text {s}^\star ) = 0.1$. In the same manner, we define ${k_\text {s}^\star }^\prime$ such that $P_{\ge }({k_\text {s}^\star }^\prime ) = 0.1$ in network $G^\prime$. To calculate *J* and $\tau _K$, we use the nodes belonging to $k$-shells with $k_\text {s}\ge k_\text {s}^\star$ in *G* and the nodes belonging to $k$-shells with $k_\text {s}\ge {k_\text {s}^\star }^\prime$ in $G^\prime$ without duplication of the nodes. There are several remarks. First, it may hold that $k_\text {s}^\star \ne {k_\text {s}^\star }^\prime$. Second, the value of ${k_\text {s}^\star }^\prime$ varies from one combination of a run of shuffling and community detection to another. Third, as in the case of the Facebook 2 data set, ${k_\text {s}^\star }^\prime$ sometimes does not even exist. In such a case, we set ${k_\text {s}^\star }^\prime = D$ and select all the nodes belonging to the innermost $k$-shell although they constitute more than 10% of the nodes in the network. Fourth, additional tests using different threshold percentages, 5% and 20%, instead of 10%, did not qualitatively change the results. Fifth, while the Jaccard score simply compares the nodes belonging to two sets, the generalised Kendall’s tau, $\tau _K$ compares ranked sets. In our case, the node’s rank is equivalent to the $k_\text {s}$ value.

Given two sets ${\mathcal {A}}$ and ${\mathcal {B}}$, the Jaccard score quantifies their overlap and is given by

3 $$\begin{aligned} J({\mathcal {A}},{\mathcal {B}}) = \dfrac{|{\mathcal {A}} \cap {\mathcal {B}}|}{|{\mathcal {A}} \cup {\mathcal {B}}|}\,. \end{aligned}$$

The Jaccard score ranges between 0 and 1. A value of 1 indicates the complete overlap between the two sets (i.e., the sets are the same), whereas a value of 0 indicates that the sets are completely different.

The generalised Kendall’s tau, $\tau _K$, measures the consistency between two rankings by assigning penalties to pairs of elements on which the two rankings disagree^65,66^. Given two sets ${\mathcal {A}}$ and ${\mathcal {B}}$ having $m_A$ and $m_B$ elements, respectively, consider their associated ranking functions ${\mathcal {X}}$ and ${\mathcal {Y}}$. We denote with $(z_1, z_2)$ an arbitrary pair of elements of ${\mathcal {A}} \cup {\mathcal {B}}$. We assign a penalty $K_{z_1,z_2}({\mathcal {X}},{\mathcal {Y}}) = 1$ to $(z_1, z_2)$ if (a) the rankings of the two elements within each set are different (i.e., ${\mathcal {X}}(z_1) \gtrless {\mathcal {X}}(z_2)$ and ${\mathcal {Y}}(z_1) \lessgtr {\mathcal {Y}}(z_2)$), (b) the element with the higher rank in one set is missing in the other set, i.e., ${\mathcal {X}}(z_1) > {\mathcal {X}}(z_2)$ and $z_1 \notin {\mathcal {B}}$ (or ${\mathcal {X}}(z_2) > {\mathcal {X}}(z_1)$ and $z_2 \notin {\mathcal {B}}$), or (c) both elements belong to one set each, which is not the same set, i.e., $z_1 \notin {\mathcal {B}}$ and $z_2 \notin {\mathcal {A}}$ (and vice-versa). In all the other cases $K_{z_1,z_2}({\mathcal {X}},{\mathcal {Y}}) = 0$, such that we do not penalise the $(z_1, z_2)$ pair. Finally, we sum the penalties over all the possible pairs of elements and normalise it, thus obtaining the generalised Kendall’s tau:

4 $$\begin{aligned} \tau _K({\mathcal {X}},{\mathcal {Y}}) = 1 - \frac{1}{m_A m_B} \sum _{z_1, z_2 \in {\mathcal {A}} \cup {\mathcal {B}}} K_{z_1,z_2}({\mathcal {X}},{\mathcal {Y}}). \end{aligned}$$

Index $\tau _K$ ranges between 0 and 1. If $\tau _K = 1$, the two rankings are completely coherent. If $\tau _K = 0$, the two sets ${\mathcal {A}}$ and ${\mathcal {B}}$ have no pair of elements on which rankings ${\mathcal {X}}$ and ${\mathcal {Y}}$ are coherent. The above formulation of the Kendall’s tau is the so-called optimistic approach^65^. This means that we do not penalise the case in which a pairs of elements is present in one set and not in the other set.

### Community detection methods

We considered two methods for community detection. The first is the Louvain method (Lvn)^39^, which is a heuristic greedy multiscale method that approximately maximises the modularity function. Given a network with *N* nodes distributed among $N_\text {c}$ communities, the modularity, *Q*, reads

5 $$\begin{aligned} Q = \dfrac{1}{2L} \sum _{i,j = 1}^N \left[ a_{i,j} - \dfrac{k_i k_j}{2L} \right] \delta \bigl (g(i),g(j)\bigr ) \,, \end{aligned}$$

where $a_{i,j}$ is the element of the network’s adjacency matrix *A*; *g*(*i*) is the community to which the *i*-th node belongs ($1 \le g(i) \le N_\text {c}$), and $\delta \bigl (g(i),g(j)\bigr )$ is the Kronecker delta. A large value of *Q* implies a good partitioning. The Louvain method seeks the partitioning that maximises the modularity. Note that we obtain $Q \approx 0$ for random assignment of nodes to communities and that we obtain $Q \approx 1$ when the network is made of perfectly disjoint communities.

The other community detection method that we used is the stochastic block model^67^. It uses the probabilities ${\mathcal {P}} = \{ p_{C_i,C_j} \}$ with which there exists an edge (*a*, *b*) connecting an arbitrarily selected node *a* in community $C_i$ (i.e., $g(a) = C_i$) and an arbitrarily selected node *b* in community $C_j$ (i.e., $g(b) = C_j$). Different instances of probabilities ${\mathcal {P}}$ allow the description of different mixing patterns. When the diagonal entries of ${\mathcal {P}}$ predominate, we obtain the most usual community structure, whereas other instances yield other structures such as bipartite or core-periphery structure.

To find the optimal partition, one maximises the likelihood function with respect to $\{ p_{C_i,C_j} \}$ corresponding to the partitioning ${\mathcal {C}} = \{ C_i \}$, where $i,j \in 1,\ldots ,N_\text {c}$. The unnormalised log-likelihood, ${\mathfrak {L}}$, with which a partition of network *G* into $N_\text {c}$ communities, ${\mathcal {C}}$, is reproduced reads

6 $$\begin{aligned} {\mathfrak {L}} \bigl ( G \, \bigl \vert \, {\mathcal {C}} \bigr . \bigr ) = \sum _{i,j = 1}^{N_\text {c}} e_{ij} \, \log \left( \frac{e_{ij}}{m_i \, m_j} \right) \,, \end{aligned}$$

where $e_{ij}$ is the number of edges connecting community $C_i$ and community $C_j$, and $m_i$ is the number of nodes belonging to $C_i$.

The above formulation, however, has one major limitation: it assumes that the degrees of the nodes are distributed according to a Poisson-like function. To account for the degrees’ heterogeneity, Karrer et al. have implemented the so-called degree corrected stochastic block model, in which the expected degree of each node is kept constant via the introduction of additional parameters^40^. Let $e_i$ be the sum of the node’s degree over all nodes in community $C_i$. Then, the unnormalised log-likelihood for the degree-corrected stochastic block model reads

7 $$\begin{aligned} {\mathfrak {L}}_{\text {DC}} \bigl ( G \, \bigl \vert \, {\mathcal {C}} \bigr . \bigr ) = \sum _{i,j = 1}^{N_\text {c}} e_{ij} \, \log \left( \frac{e_{ij}}{e_i \, e_j} \right) \,. \end{aligned}$$

Equations (6) and (7) depend on the number of communities $N_\text {c}$. Because the value of $N_\text {c}$ is not known a priori, it is inferred through the minimisation of a quantity called the description length. The minimum description length principle describes how much a model compresses the data and allows us to find the optimal number of communities while avoiding overfitting^68^. In the present work we use the degree-corrected stochastic block model and its implementation available in the Python Graph-tool package^69^, which we refer to as SBM for brevity.

## Supplementary information

Supplementary Information.

## Data Availability

The data sets on Cookpad™analysed in the current study are not publicly available due to exclusive ownership of Cookpad Limited. All the other data sets are available from the corresponding repositories listed in the bibliography.
