# Supplementary material for: Interplay between k-core and community structure in complex networks
Source: Sci Rep. 2020 Sep 7;10:14702. doi: 10.1038/s41598-020-71426-8 (PMC7477593; doi:10.1038/s41598-020-71426-8)
Supplement: Supplementary file 1 — Supplementary Information. [file 41598_2020_71426_MOESM1_ESM.pdf]

Supplementary Materials for the manuscript entitled: Interplay between k-core and community structure in complex networks

Irene Malvestio, Alessio Cardillo, & Naoki Masuda

1 Comparison between the original and shuffled networks

In this section, we provide a detailed characterisation of the k-core decomposition of the shuffled networks for all the empirical networks. Supplementary Figure S1 shows the survival function of the probability distribution of the k-shell index, $P_{\geq}(k_s)$, of the original networks and their shuffled counterparts (`deg`, `commA`, and `commB`). The figure indicates that `commA` and `commB` produce k-shell distributions that are more similar to the original ones, compared to `deg`, in particular when `commA` or `commB` is combined with `SBM`. This result also holds true for the Greece and Spain networks of Cookpad where, contrarily to the other data sets, the `deg` networks have a degeneracy, D, higher than the original networks.

In Supplementary Table S1, we report the values of the four indicators used for comparing the k-core decomposition between the original and shuffled networks. In particular, we report the average value and standard deviation of the relative difference $\Delta X = |X(G) - X(G')| / X(G)$ where X is either the average k-shell index, $\langle k_s \rangle$, or D. In the same table, we also report the values of the Jaccard score, J, and Kendall's tau, τ_K, calculated for the set of nodes belonging to the innermost k-shells (see the main text for the details of the methods). We notice that, in general, `commB-SBM` yields the smallest values of $\Delta\langle k_s \rangle$ and ΔD and the largest values of J and τ_K; confirming its good performances in reconstructing the k-core decomposition of the original network. Supplementary Figure S2 provides an overview of the performances of each shuffling method. Figure 2 in the main text is a projection of the information contained in Supplementary Fig. S2.

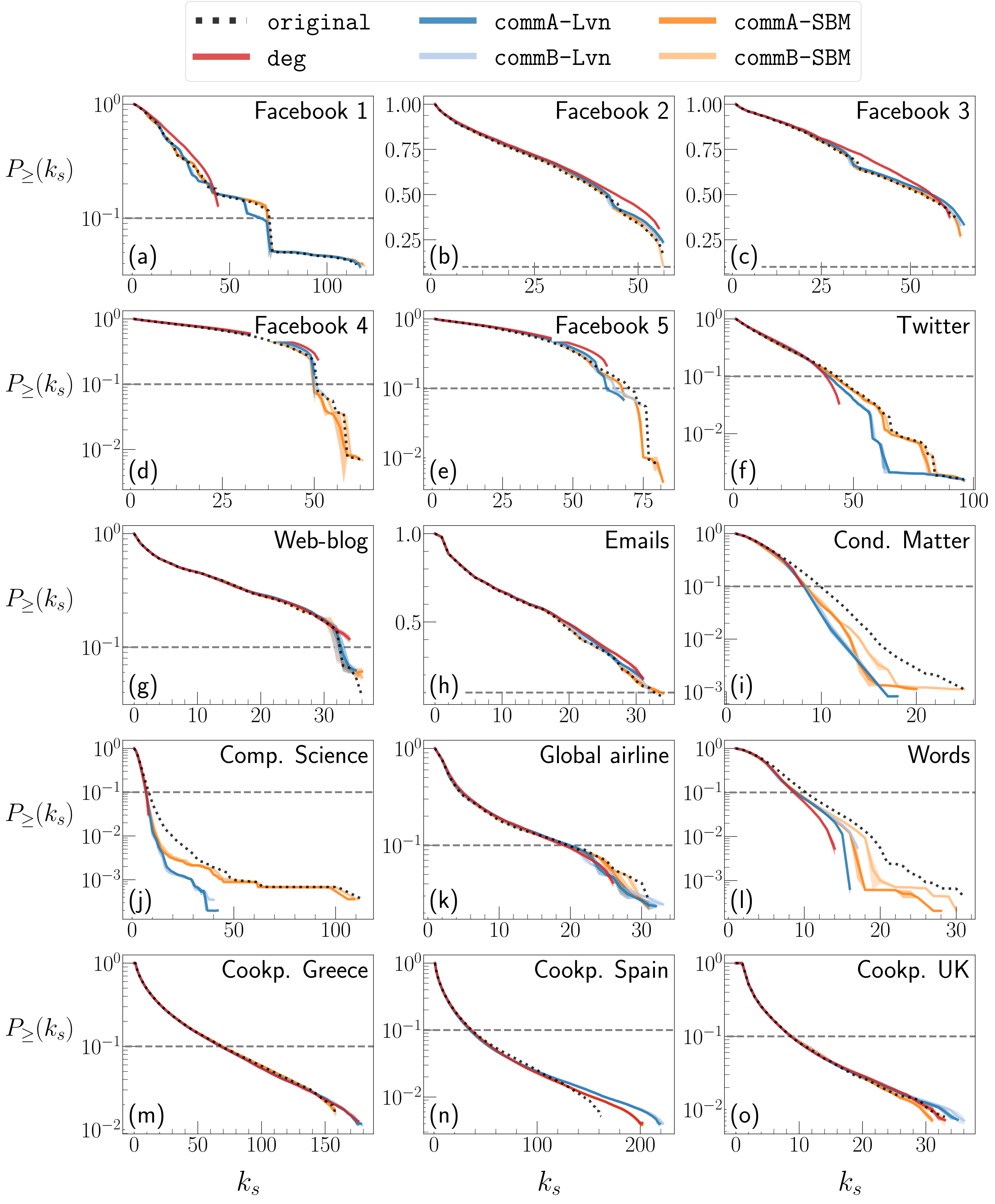

Supplementary Figure S1: Survival function of the probability distribution of the k-shell index, $P_{\geq}(k_s)$, as a function of k_s for the empirical network (dotted lines) and shuffled networks (solid lines). Each panel corresponds to a data set. The horizontal dashed lines represent $P_{\geq}(k_s) = 0.1$. The results shown are averages over 10 different runs of each shuffling method, and the shaded areas (when visible) represent the standard deviations.

Data set	Indicator	deg	commA-Lvn	commB-Lvn	commA-SBM	commB-SBM
Facebook 1	$\Delta\langle k_s\rangle$	0.132 ± 0.002	0.011 ± 0.001	0.007 ± 0.003	0.004 ± 0.002	0.002 ± 0.001
	ΔD	0.622 ± 0.004	0.004 ± 0.006	0.009 ± 0.007	0.014 ± 0.006	0.017 ± 0.007
	J	0.340 ± 0.025	0.512 ± 0.009	0.515 ± 0.004	0.722 ± 0.212	0.787 ± 0.207
	τ_K	0.884 ± 0.005	0.971 ± 0.014	0.974 ± 0.012	0.963 ± 0.015	0.980 ± 0.018
Facebook 2	$\Delta\langle k_s\rangle$	0.043 ± 0.000	0.020 ± 0.003	0.011 ± 0.001	0.006 ± 0.000	0.002 ± 0.001
	ΔD	0.018 ± 0.000	0.007 ± 0.009	0.018 ± 0.000	0.018 ± 0.000	0.012 ± 0.008
	J	0.515 ± 0.008	0.664 ± 0.038	0.695 ± 0.014	0.685 ± 0.021	0.737 ± 0.064
	τ_K	1.000 ± 0.000	1.000 ± 0.000	1.000 ± 0.000	1.000 ± 0.000	1.000 ± 0.000
Facebook 3	$\Delta\langle k_s\rangle$	0.019 ± 0.004	0.025 ± 0.002	0.017 ± 0.003	0.007 ± 0.003	0.002 ± 0.001
	ΔD	0.035 ± 0.006	0.017 ± 0.005	0.011 ± 0.007	0.006 ± 0.008	0.000 ± 0.000
	J	0.788 ± 0.009	0.853 ± 0.009	0.856 ± 0.027	0.840 ± 0.066	0.918 ± 0.017
	τ_K	1.000 ± 0.000	1.000 ± 0.000	1.000 ± 0.000	1.000 ± 0.000	1.000 ± 0.000
Facebook 4	$\Delta\langle k_s\rangle$	0.044 ± 0.003	0.012 ± 0.002	0.005 ± 0.002	0.003 ± 0.001	0.002 ± 0.001
	ΔD	0.203 ± 0.006	0.206 ± 0.000	0.165 ± 0.008	0.027 ± 0.010	0.005 ± 0.007
	J	0.247 ± 0.017	0.531 ± 0.186	0.744 ± 0.015	0.837 ± 0.005	0.951 ± 0.016
	τ_K	0.818 ± 0.011	0.584 ± 0.154	0.535 ± 0.027	0.865 ± 0.008	0.959 ± 0.014
Facebook 5	$\Delta\langle k_s\rangle$	0.030 ± 0.002	0.002 ± 0.001	0.002 ± 0.001	0.001 ± 0.001	—
	ΔD	0.237 ± 0.005	0.169 ± 0.006	0.098 ± 0.004	0.017 ± 0.006	—
	J	0.406 ± 0.023	0.619 ± 0.004	0.664 ± 0.004	0.712 ± 0.015	—
	τ_K	0.724 ± 0.016	0.699 ± 0.016	0.721 ± 0.011	0.881 ± 0.014	—
Twitter	$\Delta\langle k_s\rangle$	0.055 ± 0.000	0.028 ± 0.000	0.030 ± 0.000	0.014 ± 0.000	0.008 ± 0.000
	ΔD	0.542 ± 0.000	0.005 ± 0.005	0.007 ± 0.005	0.011 ± 0.007	0.007 ± 0.005
	J	0.540 ± 0.001	0.641 ± 0.008	0.637 ± 0.005	0.834 ± 0.003	0.881 ± 0.011
	τ_K	0.580 ± 0.004	0.749 ± 0.002	0.755 ± 0.002	0.884 ± 0.002	0.941 ± 0.002
Web-blog	$\Delta\langle k_s\rangle$	0.005 ± 0.002	0.004 ± 0.003	0.004 ± 0.002	0.007 ± 0.004	0.003 ± 0.002
	ΔD	0.069 ± 0.014	0.056 ± 0.012	0.042 ± 0.014	0.022 ± 0.011	0.008 ± 0.013
	J	0.416 ± 0.009	0.780 ± 0.021	0.775 ± 0.022	0.853 ± 0.021	0.920 ± 0.017
	τ_K	0.794 ± 0.018	0.619 ± 0.061	0.646 ± 0.038	0.651 ± 0.068	0.664 ± 0.070
Emails	$\Delta\langle k_s\rangle$	0.011 ± 0.006	0.005 ± 0.002	0.003 ± 0.001	0.005 ± 0.003	0.002 ± 0.001
	ΔD	0.103 ± 0.015	0.103 ± 0.015	0.065 ± 0.012	0.024 ± 0.012	0.029 ± 0.000
	J	0.422 ± 0.044	0.454 ± 0.035	0.671 ± 0.069	0.701 ± 0.027	0.845 ± 0.057
	τ_K	0.908 ± 0.008	0.895 ± 0.008	0.864 ± 0.023	0.855 ± 0.011	0.842 ± 0.048
Cond. Matter	$\Delta\langle k_s\rangle$	0.123 ± 0.001	0.111 ± 0.001	0.113 ± 0.001	0.117 ± 0.001	0.113 ± 0.001
	ΔD	0.680 ± 0.000	0.284 ± 0.012	0.296 ± 0.020	0.244 ± 0.022	0.016 ± 0.020
	J	0.395 ± 0.006	0.423 ± 0.013	0.481 ± 0.008	0.548 ± 0.013	0.615 ± 0.005
	τ_K	0.521 ± 0.018	0.415 ± 0.025	0.479 ± 0.016	0.623 ± 0.013	0.685 ± 0.008
Comp. Science	$\Delta\langle k_s\rangle$	0.171 ± 0.004	0.153 ± 0.000	0.159 ± 0.000	0.146 ± 0.000	0.144 ± 0.000
	ΔD	0.933 ± 0.004	0.641 ± 0.006	0.651 ± 0.004	0.047 ± 0.007	0.023 ± 0.004
	J	0.374 ± 0.069	0.488 ± 0.002	0.456 ± 0.002	0.504 ± 0.003	0.493 ± 0.002
	τ_K	0.371 ± 0.358	0.688 ± 0.003	0.673 ± 0.002	0.686 ± 0.002	0.711 ± 0.002
Global airline	$\Delta\langle k_s\rangle$	0.023 ± 0.001	0.004 ± 0.003	0.008 ± 0.003	0.009 ± 0.003	0.010 ± 0.002
	ΔD	0.161 ± 0.000	0.013 ± 0.016	0.035 ± 0.017	0.010 ± 0.015	0.003 ± 0.010
	J	0.766 ± 0.011	0.826 ± 0.010	0.824 ± 0.039	0.852 ± 0.016	0.933 ± 0.008
	τ_K	0.661 ± 0.016	0.831 ± 0.006	0.846 ± 0.014	0.872 ± 0.020	0.932 ± 0.006
Words	$\Delta\langle k_s\rangle$	0.118 ± 0.000	0.105 ± 0.000	0.108 ± 0.000	0.107 ± 0.000	0.102 ± 0.000
	ΔD	0.552 ± 0.010	0.484 ± 0.000	0.458 ± 0.013	0.097 ± 0.000	0.032 ± 0.000
	J	0.473 ± 0.003	0.575 ± 0.001	0.575 ± 0.002	0.592 ± 0.001	0.675 ± 0.002
	τ_K	0.668 ± 0.003	0.740 ± 0.001	0.758 ± 0.003	0.794 ± 0.003	0.815 ± 0.001
Cookpad – Greece	$\Delta\langle k_s\rangle$	0.002 ± 0.000	0.001 ± 0.000	0.002 ± 0.000	0.002 ± 0.001	0.000 ± 0.000
	ΔD	0.110 ± 0.003	0.113 ± 0.007	0.129 ± 0.003	0.016 ± 0.006	0.004 ± 0.003
	J	0.846 ± 0.003	0.865 ± 0.003	0.892 ± 0.004	0.941 ± 0.002	0.964 ± 0.002
	τ_K	0.850 ± 0.001	0.871 ± 0.001	0.894 ± 0.001	0.943 ± 0.002	0.968 ± 0.004
Cookpad – Spain	$\Delta\langle k_s\rangle$	0.002 ± 0.000	0.005 ± 0.000	0.005 ± 0.000	0.002 ± 0.000	0.002 ± 0.000
	ΔD	0.240 ± 0.006	0.346 ± 0.006	0.362 ± 0.005	0.238 ± 0.005	0.234 ± 0.006
	J	0.762 ± 0.006	0.872 ± 0.004	0.903 ± 0.003	0.760 ± 0.005	0.762 ± 0.006
	τ_K	0.837 ± 0.001	0.890 ± 0.001	0.907 ± 0.000	0.837 ± 0.001	0.837 ± 0.001
Cookpad – UK	$\Delta\langle k_s\rangle$	0.001 ± 0.001	0.005 ± 0.001	0.012 ± 0.001	0.001 ± 0.001	0.001 ± 0.000
	ΔD	0.030 ± 0.014	0.027 ± 0.016	0.085 ± 0.012	0.076 ± 0.015	0.012 ± 0.015
	J	0.754 ± 0.010	0.781 ± 0.007	0.827 ± 0.005	0.835 ± 0.003	0.886 ± 0.006
	τ_K	0.826 ± 0.003	0.852 ± 0.003	0.893 ± 0.002	0.886 ± 0.002	0.922 ± 0.002

Supplementary Table S1: Average and standard deviation of the four indicators characterising the k-core decomposition. In the cells with missing values, the shuffling method did not converge.

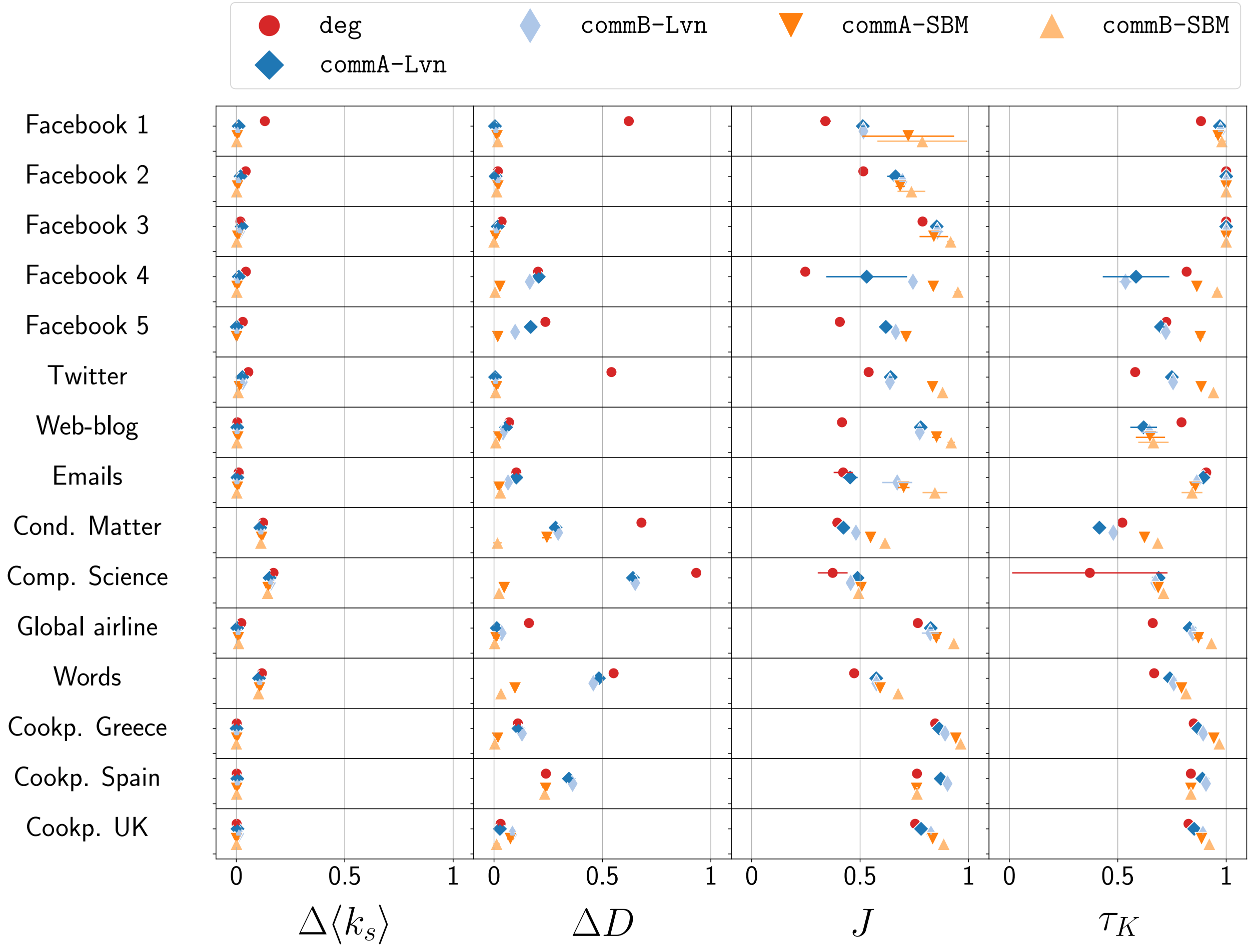

Supplementary Figure S2: Graphical summary of the values reported in Supplementary Table S1. For each pair of an empirical network and indicator, we show the value of the indicator for each shuffling method. The error bars represent the standard deviation.

2 Effects of changing the resolution of the Louvain method

A pitfall of the Louvain method is its propensity to merge small communities due to the existence of a lower bound in the size of the communities (*i.e.*, the modularity's resolution limit) [1,2]. However, communities of empirical networks may not have a typical size, and small communities coexist with large ones in general. A method for mitigating the resolution limit is to introduce a resolution parameter $r \in (0, 1]$ into the Louvain algorithm [3]. By tuning r, it is possible to vary the resolution scale of the detected communities, spanning from large (*i.e.*, $r \sim 1$) to small (*i.e.*, $r \sim 0$) communities. We denote the Louvain method using a different resolution r by `LvnR`.

Figure 2 in the main text shows that `commB` combined with the communities found by the `SBM` reproduces the tail of $P_{\geq}(k_s)$ of the empirical networks most accurately. Table 1 indicates that `SBM` tends to find more communities than `Lvn`, although there are exceptions. To understand whether the different number of communities found by `SBM` and `Lvn` is the reason behind their different performances, we extracted the communities using `LvnR` with $r \in \{0.1, 0.3, 0.5\}$. For the sake of brevity, we only discuss the results for $r = 0.3$ in the following text. However, we have verified that the results for the other values of r are similar.

In Supplementary Table S2 we show, for all the data sets considered, the number of communities, N_c, and the modularity, Q, corresponding to the community structure found using `Lvn`, `SBM`, and `LvnR` with $r = 0.3$. With `LvnR`, the number of communities is similar or even larger than that obtained with `SBM`. Supplementary Figures S3 and S4 show $P_{\geq}(k_s)$ plotted against k_s and the four indicators used for comparing the innermost k-shells, respectively, including the results obtained with `LvnR`. These figures indicate that using $r = 0.3$ as opposed to $r = 1$ improves the ability of the Louvain algorithm to mimic the structure of the k-shell. In Supplementary Fig. S5 we summarise the performances of the different shuffling methods including `LvnR`.

Although `LvnR` mimics the k-shell features better than `SBM` for some data sets and indicators (approximately around the 20% of the cases), `SBM` still attains the highest success ratio for each indicator, f_X. The few cases for which `commB-LvnR` does better than `commB-SBM` are the networks for which the difference between the empirical $P_{\geq}(k_s)$ and $P_{\geq}(k_s)$ obtained from the `deg` shuffling apparently looks small, such as the Emails and Cookpad UK networks. In these networks, the differences between the $P_{\geq}(k_s)$ of the networks obtained using `commB-LvnR` and `commB-SBM` are also small. By contrast, other data sets such as Condensed Matter, Computer Science, and Words show bigger differences between their $P_{\geq}(k_s)$ and that obtained using the `deg` method. In these data sets, `commB-SBM` is much better than `commB-LvnR`, although `commB-LvnR` is better than `commB-Lvn`. Overall, these results suggest that the increase in the number of communities enabled by a small r does not lead to reconstruction of the k-shell structure with a better accuracy than `SBM` does.

Data set	N_c^{Lvn}	Q^{Lvn}	N_c^{SBM}	Q^{SBM}	N_c^{LvnR}	Q^{LvnR}
Facebook 1	16	0.835	62	0.551	29	0.819
Facebook 2	19	0.419	198	0.158	67	0.348
Facebook 3	8	0.436	87	0.139	36	0.294
Facebook 4	10	0.438	274	0.193	72	0.381
Facebook 5	18	0.470	547	0.172	92	0.417
Twitter	73	0.808	510	0.511	136	0.779
Web-blogs	275	0.426	17	0.076	331	0.150
Emails	26	0.410	33	0.232	61	0.350
Cond. Matter	619	0.730	203	0.633	716	0.718
Comp. Science	209	0.822	676	0.726	438	0.812
Global airline	26	0.665	40	0.311	55	0.542
Words	378	0.759	548	0.583	523	0.747
Cookpad Greece	40	0.166	76	0.020	365	0.067
Cookpad Spain	262	0.270	90	0.035	501	0.164
Cookpad UK	199	0.350	8	0.114	320	0.314

Supplementary Table S2: Summary of the properties of the community structures of the data sets analysed using different community detection methods. For each pair (network, method) we computed the number of communities, N_c, and the value of the modularity Q. Each pair of columns accounts for a different method, namely: Louvain (`Lvn`), stochastic block model (`SBM`), and Louvain with a resolution parameter $r = 0.3$ (`LvnR`).

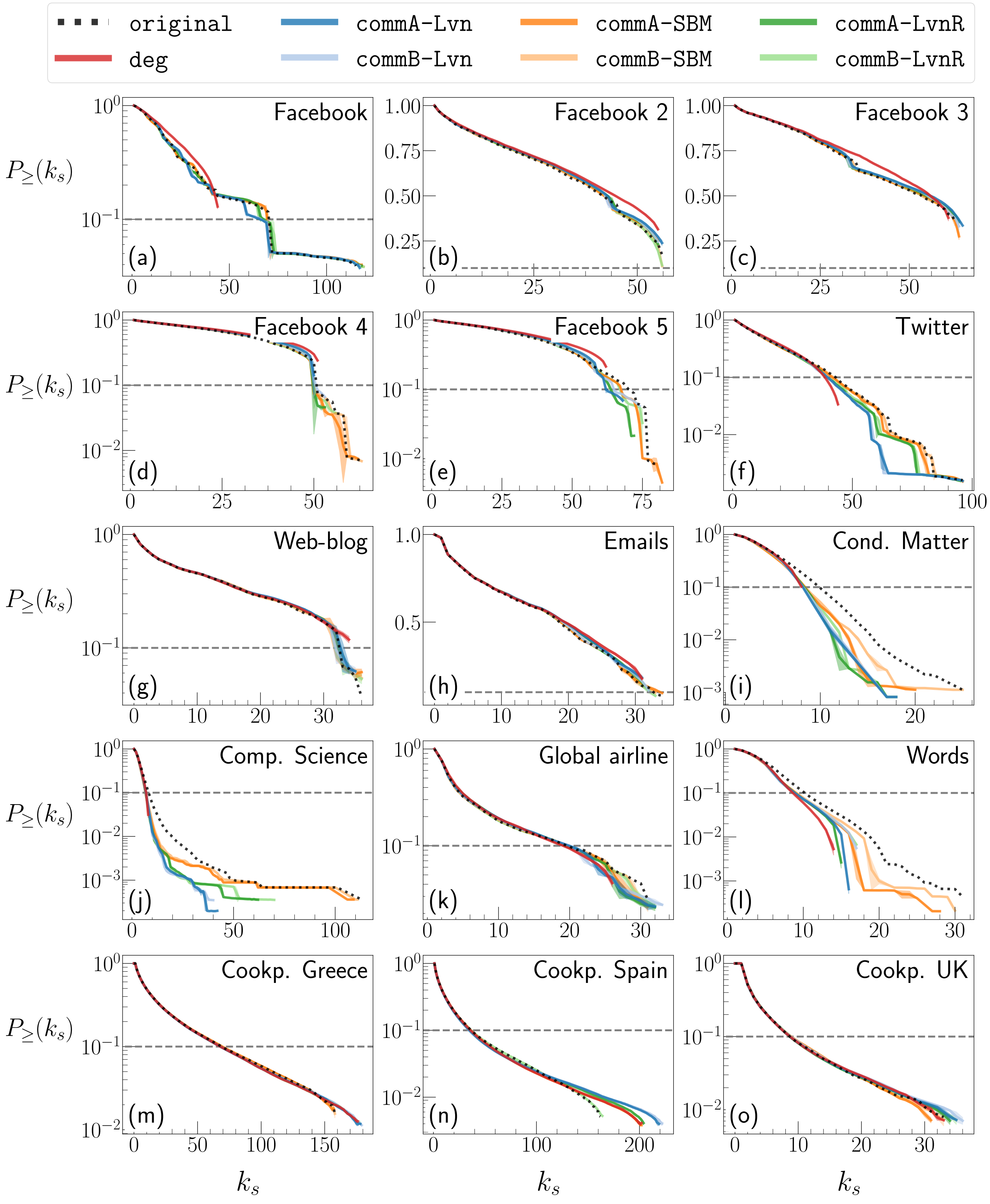

Supplementary Figure S3: Survival function of the probability distribution of the k-shell index, $P_{\geq}(k_s)$, as a function of k_s for all the cases considered in Fig. 1 plus the cases in which we identify communities using the Louvain method with a resolution parameter of $r = 0.3$. See the caption or Supplementary Fig. S1 for notations and legends.

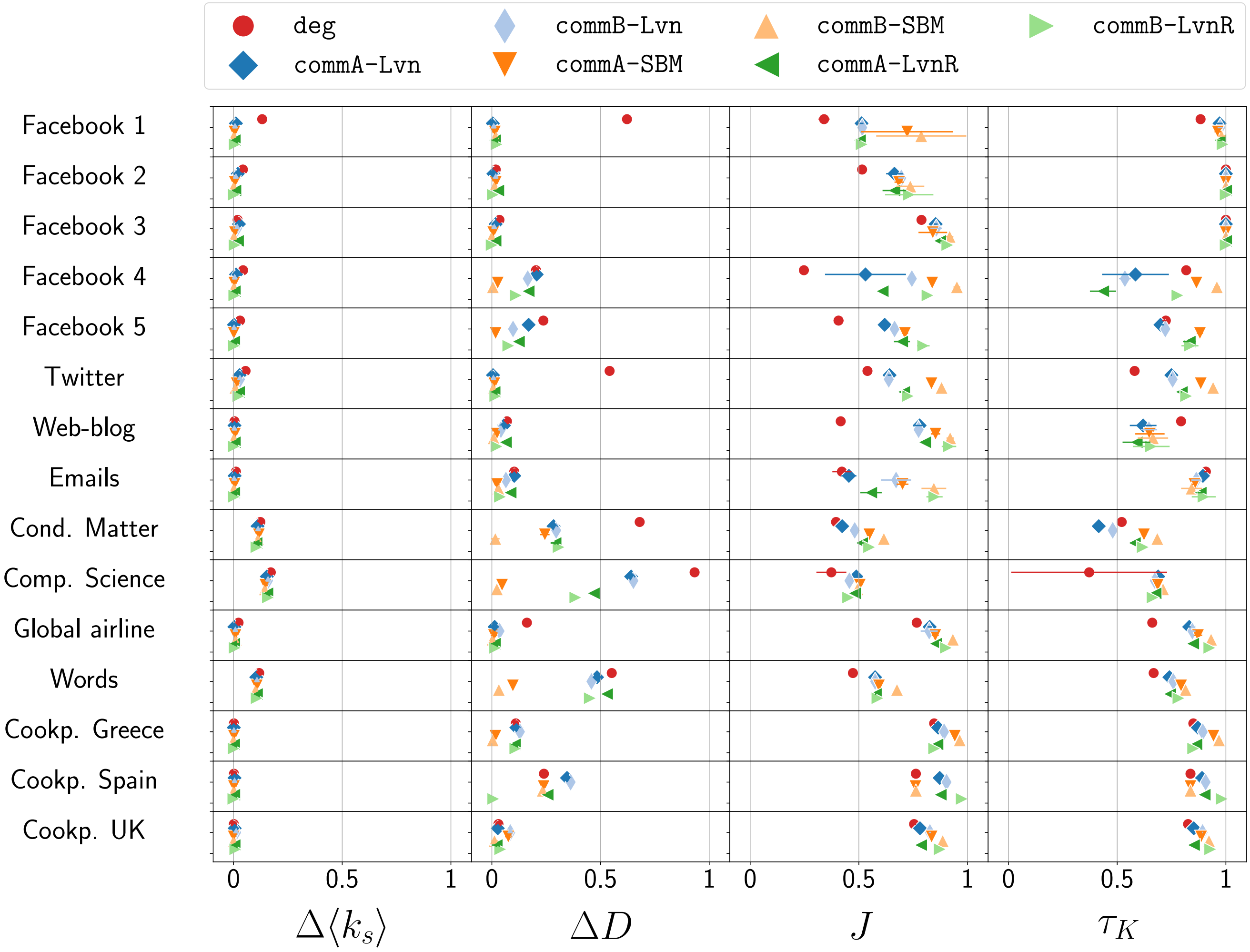

Supplementary Figure S4: Graphical summary of the average and standard deviation of the four indicators characterising the k-core decomposition. In addition to the information displayed in Supplementary Fig. S2, we consider the community structure detected by the Louvain method with $r = 0.3$ (LvnR). See the caption or Supplementary Fig. S2 for notations and legends.

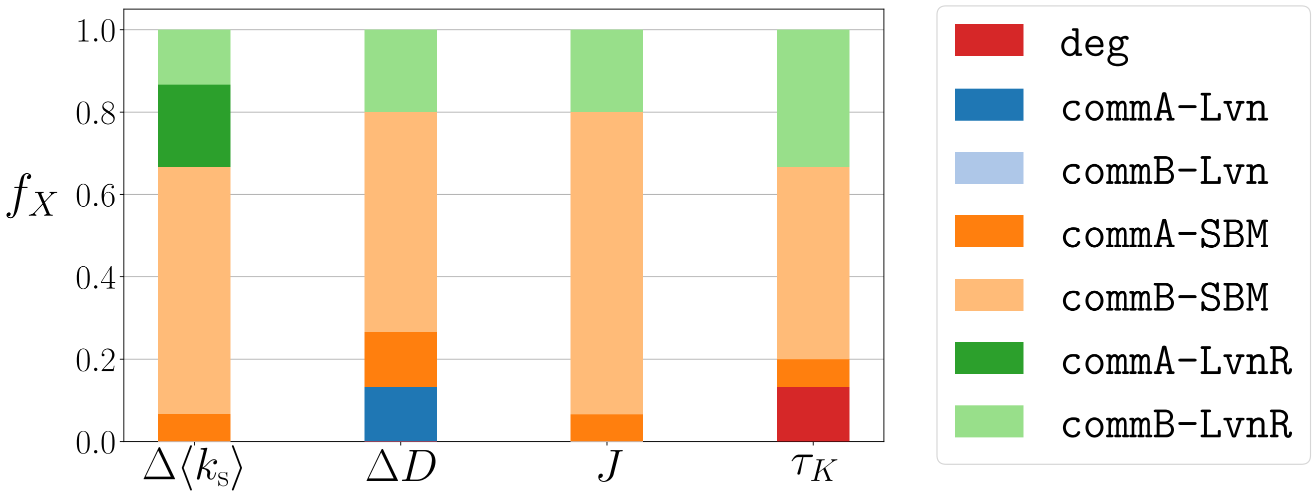

Supplementary Figure S5: Summary of the performances of the different shuffling methods in reproducing the features of the k-shells according to four indicators. We report the fraction of data sets for which a given combination of the shuffling method and the community detection method yields an indicator's value closest to that for the original network. In addition to the methods considered in Fig. 2, we also consider the case of the communities extracted using the Louvain method with $r = 0.3$. See the caption or Fig. 2 for notations and legends.

To investigate which features of the SBM and Louvain algorithms are responsible for the difference in their performances, we study the average of the size of the communities to which nodes of a certain k-shell belong, $\langle s_{\mathrm{C}} \rangle$, as a function of the k-shell index, k_{s}. In Supplementary Fig. S6, we observe how $\langle s_{\mathrm{C}} \rangle$ of the communities found by the Louvain method (`Lvn`) stays nearly constant across the entire range of k_{s} values. By contrast, with `SBM`, $\langle s_{\mathrm{C}} \rangle$ monotonically decreases as k_{s} increases, such that nodes in inner k-shells tend to belong to smaller communities. Although a high resolution (*i.e.*, a small r value) in the Louvain method produces a large number of communities (see Supplementary Table S2), the behaviour of $\langle s_{\mathrm{C}} \rangle$ for `Lvn` and `LvnR` is similar, with the major difference that the value of $\langle s_{\mathrm{C}} \rangle$ is smaller for `LvnR`.

These results altogether lead us to conclude that our method combined with the community structure identified using the Louvain method with a higher resolution does not outperform our method combined with `SBM` in grasping the features of the k-core decomposition. This may be because `SBM` is capable of finding more universal mesostructures than those found by the Louvain method [4–6].

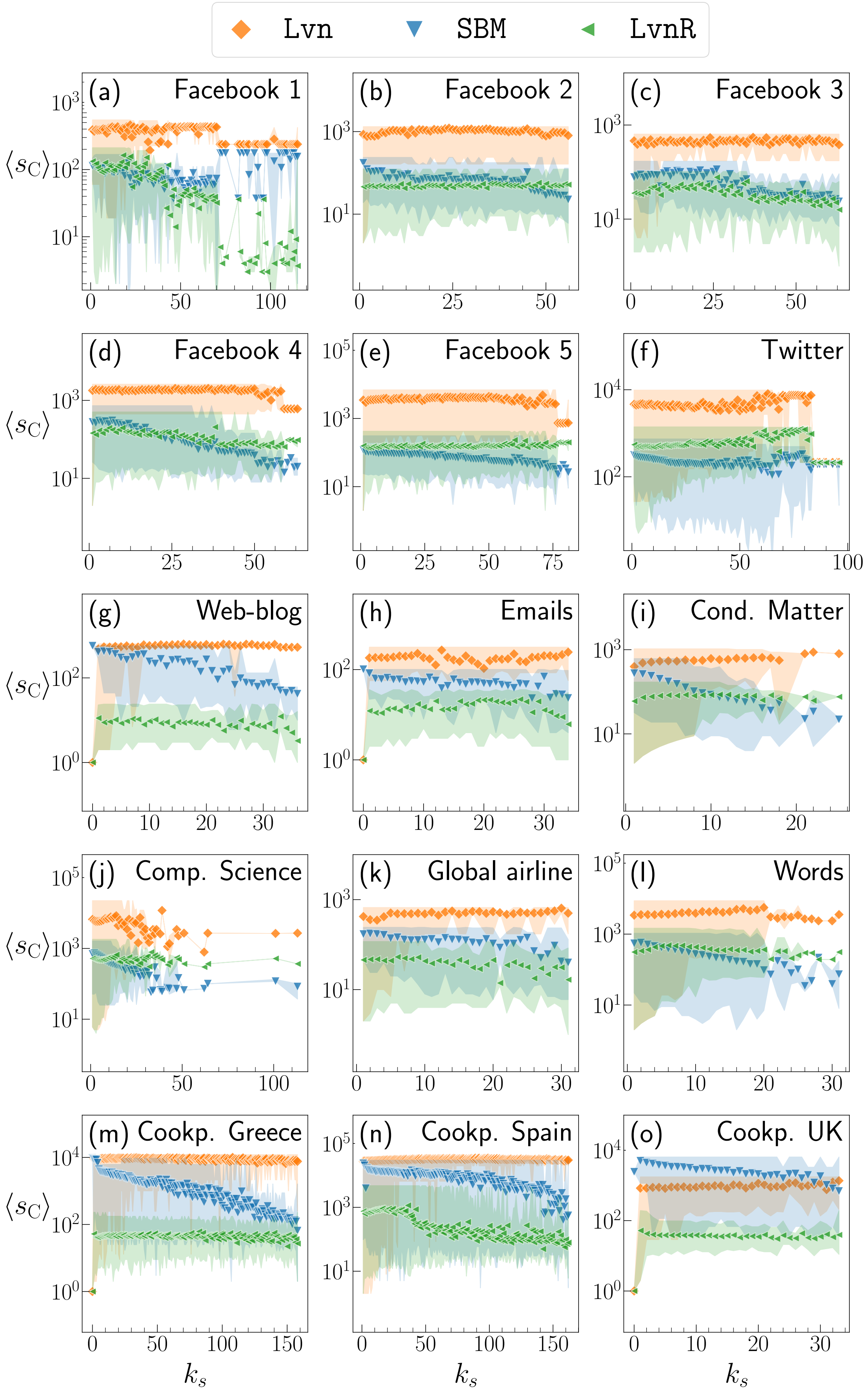

Supplementary Figure S6: Average size of the communities to which the nodes having k-shell index k_s belong to, $\langle s_C \rangle$, versus k_s. We identify the community structure using either the Louvain (`Lvn`), the stochastic block model (`SBM`), or the Louvain with higher resolution (`LvnR`) methods. The resolution parameter for (`LvnR`) is equal to $r = 0.3$. Shaded areas denote the standard deviation. Each panel accounts for a different data set.

3 The LFR model

The Lancichinetti-Fortunato-Radicchi (LFR) model generates networks where both the node's degree and the size of the communities (*i.e.*, the number of nodes belonging to a community) follow power-law distributions [7]. Such features are found in many empirical networks [8] and have led to the success of the LFR model as generator of benchmark networks to test community detection algorithms [2]. A main finding presented in the main text is that preserving the community structure of the original network in addition to the degree of each node improves the ability of the shuffling methods to mimic the k-core decomposition of the original networks. Here, to test whether or not the community structure and the degree of each node, but not a possible intricate association between the two, is sufficient for mimicking the features of k-core decomposition observed for many empirical networks, we generated networks using the LFR model and analysed their k-cores and those of the shuffled counterparts.

The LFR algorithm depends on the following parameters: the exponent, $t_1 \in [2, 3]$, of the degree distribution $P(k) \propto k^{-t_1}$; the exponent, $t_2 \in [1, 2]$, of the community's size distribution $P(S_c) \propto {S_c}^{-t_2}$; the mixing parameter, $\mu \in [0, 1]$, specifying the fraction of intra-community edges for a node. A value of $\mu = 0$ indicates that a node is connected only with nodes belonging to communities different from its own. A value of $\mu = 1$ indicates that a node is connected exclusively with nodes belonging to its own community; either one of the following: the average degree, $\langle k \rangle$, the minimum degree, $k_{\min}$, or the minimum number of communities, $\min N_{\rm c}$. This stochastic algorithm may not produce a network fulfilling all the requirements in some realisations. Therefore, we have to set the parameter values to ensure the algorithm's convergence.

To encompass a good spectrum of networks, we consider four batches of parameter sets, which are summarised in Supplementary Table S3, together with the properties of the generated networks. Each batch of parameter sets consists of a value of t_1, a value of t_2, and seven values of μ ranging from 0.1 to 0.8. We assumed $N = 10000$ nodes and used the implementation of the LFR algorithm in the NetworkX Python package [9].

For each network generated, we extracted its k-core decomposition and calculated the four indicators. We did the same for the shuffled counterparts generated using the `deg`, `commA`, and `commB` methods. In analogy to Supplementary Fig. S1, in Supplementary Figs. S7–S10 we show the survival function of the probability distribution of the k-shell index, $P_{\geq}(k_{\rm s})$, for the original LFR networks and the shuffled counterparts, one figure per each (t_1, t_2) pair. An eye inspection of Supplementary Figs. S7–S10 highlights the existence of three trends.

First, Supplementary Figs. S7 and S8 indicate that, in networks generated using the smaller t_1 values (*i.e.*, parameters batches 1 and 2 in Supplementary Table S3), the shuffled networks generated by `deg`, `commA-Lvn`, and `commB-Lvn` attain a k-core decomposition with a degeneracy, D, considerably higher than the original one. In contrast, Supplementary Figs. S9 and S10 indicate that, with the larger t_1 values (*i.e.*, parameter batches 3 and 4), we recover the same trend as that shown in Fig. 1. In other words, D for the original networks are larger than that for the shuffled networks. The difference between the original D and its shuffled counterpart seems to be influenced by the value of t_1, but not t_2 or μ.

Second, $P_{\geq}(k_{\rm s})$ for the original LFR networks mainly decreases smoothly as $k_{\rm s}$ increases, without plateaus or abrupt drops. Therefore, the k-core decomposition of LFR networks does not return any k-shell that is empty or much more populated than its adjacent k-shells. This result is in stark contrast to that for various empirical networks, *e.g.*, the Facebook 1 data set (see Supplementary Fig. S1).

Third, regardless of the values of t_1, t_2, and μ, the `commB-SBM` shuffling method produces networks with the $P_{\geq}(k_{\rm s})$ more akin to the original one than the other shuffling methods do. This result is consistent with that for the empirical networks presented in the main text.

In a nutshell, the analysis of the k-core decomposition of networks generated by the LFR model reveals that the presence of communities is not enough to justify main properties of the k-shell structure observed in the empirical networks.

LFR parameters t_1, t_2	μ	L	$k_{\min}$	$\langle k \rangle$	$k_{\max}$	$\langle k_s \rangle$	D	N_c^{Lvn}	Q^{Lvn}	N_c^{SBM}	Q^{SBM}
**Parameter batch 1**											
$t_1 = 2.2$, $t_2 = 1.5$	0.1	120893	4	24.179	3470	12.541	16	10	0.673	29	0.041
	0.2	116200	4	23.240	3380	12.201	14	4	0.497	16	-0.006
	0.3	118260	4	23.652	3199	12.188	14	7	0.458	26	-0.002
	0.4	118547	4	23.709	6309	12.287	14	7	0.234	19	-0.053
	0.5	130304	4	26.061	4481	12.548	16	7	0.250	24	-0.038
	0.6	126277	4	25.255	4607	12.967	15	10	0.164	8	-0.151
	0.8	118032	4	23.606	4028	12.263	14	10	0.162	5	-0.156
**Parameter batch 2**											
$t_1 = 2.6$, $t_2 = 2.0$	0.1	132920	8	26.584	2474	14.277	16	5	0.651	16	0.118
	0.2	129069	8	25.814	1641	14.186	15	8	0.595	22	0.139
	0.3	129024	8	25.805	1287	14.138	15	9	0.487	26	0.091
	0.4	128606	8	25.721	3305	14.015	15	7	0.222	9	-0.020
	0.5	127596	8	25.519	1504	14.105	15	8	0.227	20	0.041
	0.6	131024	8	26.205	1287	14.165	15	7	0.178	5	-0.093
	0.8	133017	8	26.603	4436	14.665	16	8	0.166	8	-0.087
**Parameter batch 3**											
$t_1 = 2.9$, $t_2 = 1.5$	0.1	315105	24	63.012	4249	35.907	37	4	0.511	14	0.115
	0.2	320836	24	64.167	2439	36.362	37	12	0.584	31	0.179
	0.3	319482	24	63.896	3732	36.234	37	10	0.359	29	0.078
	0.4	319070	24	63.814	2371	36.102	37	11	0.314	30	0.063
	0.5	321222	24	64.244	2795	36.816	38	9	0.204	27	0.029
	0.6	317738	24	63.548	2371	36.019	37	8	0.146	18	0.016
	0.8	305945	24	61.189	2246	35.049	36	9	0.109	4	-0.052
**Parameter batch 4**											
$t_1 = 3.0$, $t_2 = 2.0$	0.1	247311	20	49.462	2819	28.561	31	31	0.740	57	0.323
	0.2	246506	20	49.301	1228	27.691	28	40	0.651	69	0.331
	0.3	254822	20	50.964	1779	29.468	30	29	0.484	50	0.224
	0.4	249528	20	49.906	1131	28.457	29	37	0.387	71	0.156
	0.5	254371	20	50.874	2668	29.120	30	17	0.211	42	0.070
	0.6	243746	20	48.749	1097	28.311	29	20	0.186	50	0.073
	0.8	251094	20	50.219	3569	28.364	29	9	0.119	4	-0.053

Supplementary Table S3: Summary of the properties of the networks generated with the LFR model. For each combination of parameters t_1, t_2, and μ we report the number of edges, L, minimum degree, $k_{\min}$, average degree, $\langle k \rangle$, maximum degree, $k_{\max}$, degeneracy, D, number of communities, N_c, and modularity, Q, for communities extracted using either the Louvain (`Lvn`) or stochastic block model (`SBM`) method. All networks have $N = 10000$ nodes.

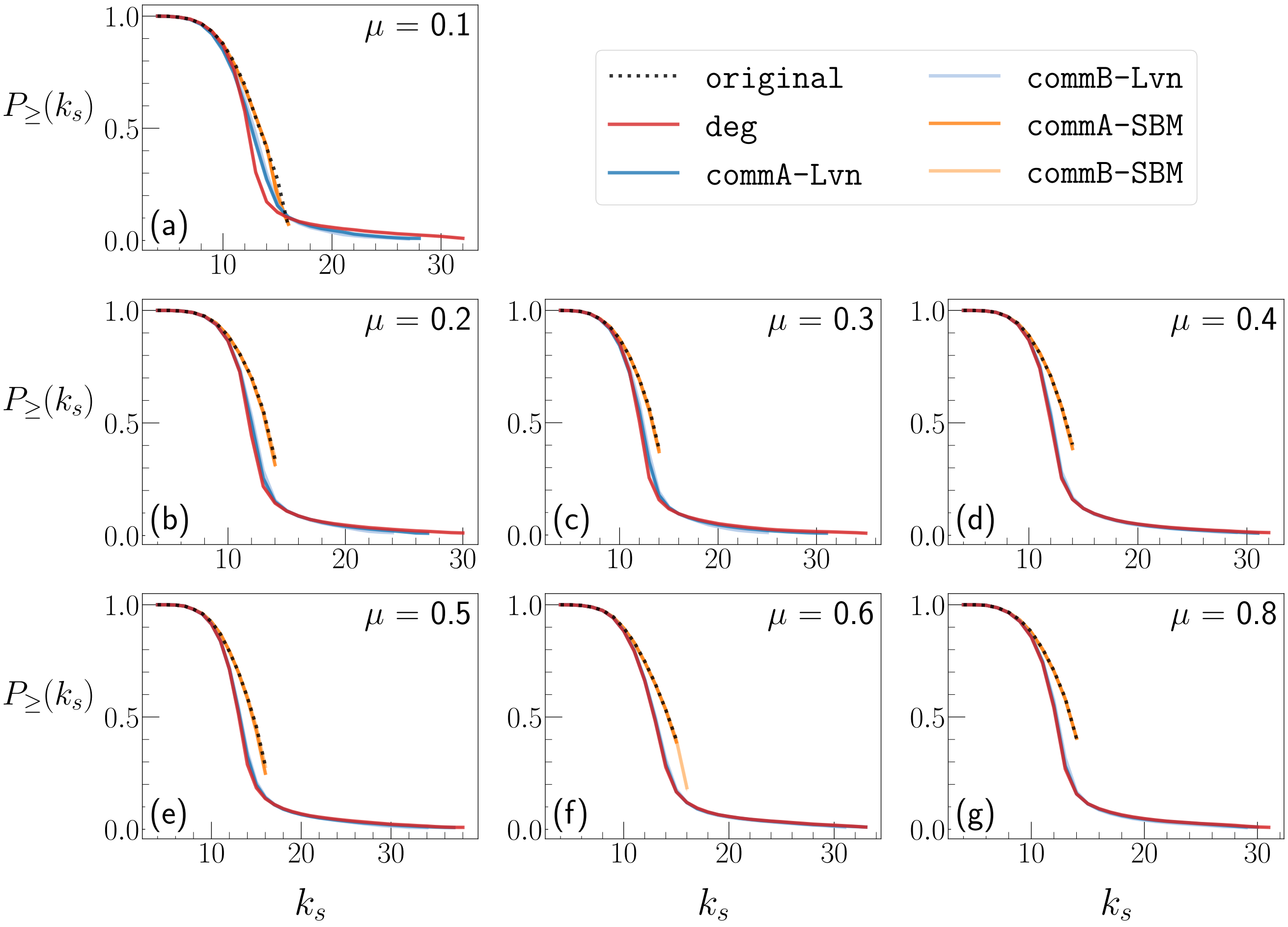

Supplementary Figure S7: Survival function of the probability distribution, $P_{\geq}(k_s)$, of the k-shell index, k_s, for the LFR networks generated using parameter batch 1 (*i.e.*, with $t_1 = 2.2$ and $t_2 = 1.5$; see Supplementary Table S3). The dotted lines correspond to the original network. The solid lines correspond to shuffled networks. Each panel corresponds to a value of μ. Shuffled results are averages over 10 realisations. The shaded area corresponds to the standard deviation. All networks have $N = 10000$ nodes.

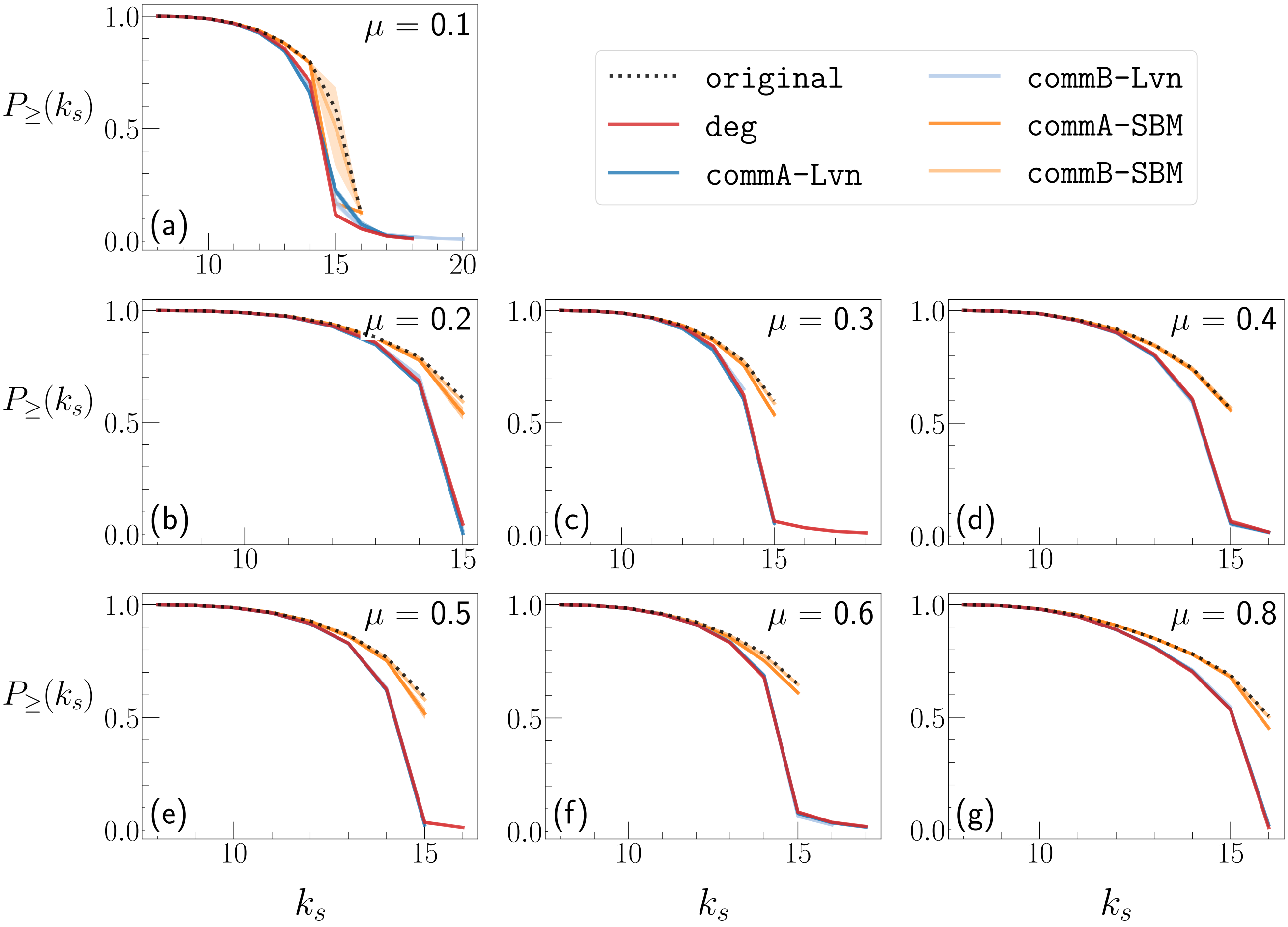

Supplementary Figure S8: Survival function of the probability distribution, $P_{\geq}(k_s)$, of the k-shell index, k_s, for the LFR networks generated using parameter batch 2 (*i.e.*, with $t_1 = 2.6$ and $t_2 = 2.0$; see Supplementary Table S3). See the caption or Supplementary Fig. S7 for notations and legends.

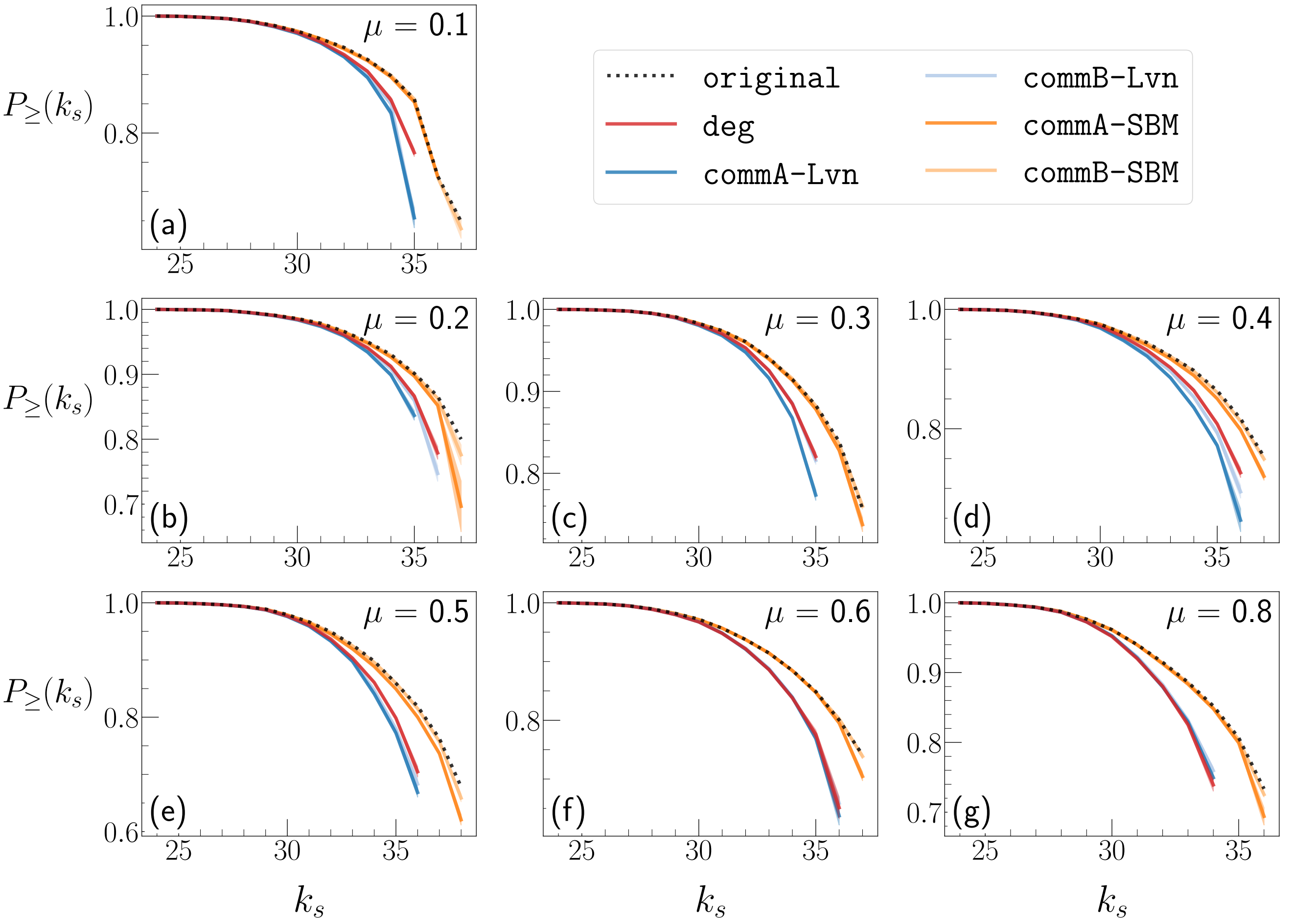

Supplementary Figure S9: Survival function of the probability distribution, $P_{\geq}(k_{\mathrm{s}})$, of the k-shell index, k_{s}, for the LFR networks generated using parameter batch 3 (*i.e.*, with $t_1 = 2.9$ and $t_2 = 1.5$; see Supplementary Table S3). See the caption or Supplementary Fig. S7 for notations and legends.

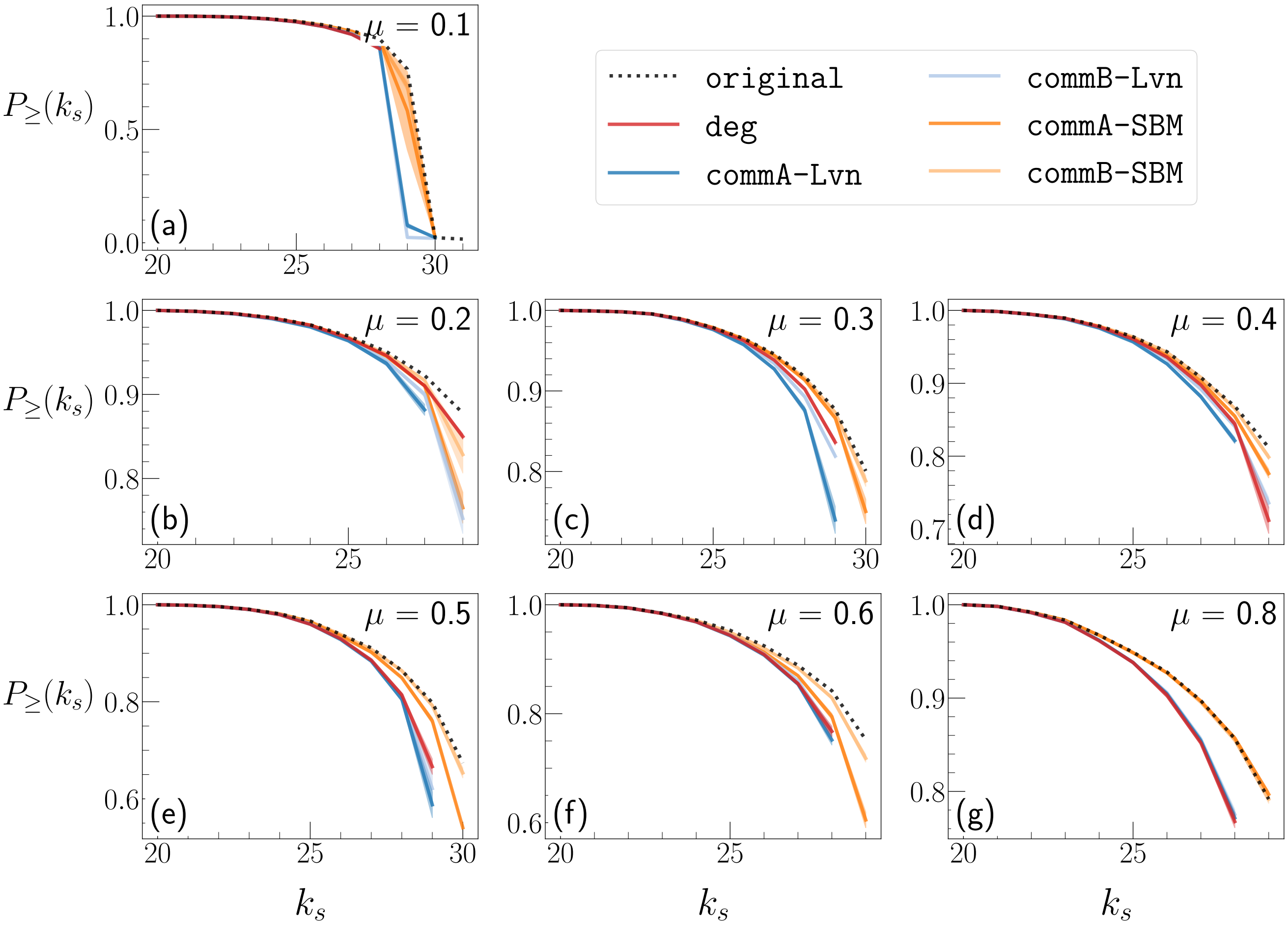

Supplementary Figure S10: Survival function of the probability distribution, $P_{\geq}(k_s)$, of the k-shell index, k_s, for the LFR networks generated using parameter batch 4 (*i.e.*, with $t_1 = 3.0$ and $t_2 = 2.0$; see Supplementary Table S3). See the caption or Supplementary Fig. S7 for notations and legends.

4 Relationship between community structure and k-core decomposition

In this section, we examine the number of communities to which the nodes in each k-shell belong, with the aim of examining whether or not those nodes are concentrated into one or a small number of communities, particularly for nodes in innermost k-shells. This analysis is similar to Supplementary Fig. S6, whereas in that case we focused on the averaged community size. Supplementary Figure S11 shows the number of distinct communities to which the nodes with a given k_{s} value belong, denoted by $n_{\mathrm{C}}(k_{\mathrm{s}})$, for all the data sets. In agreement with Fig. 3, some data sets show a strong concentration of the innermost k-shells (*i.e.*, nodes with large k_{s} values) into one or a few communities.

Next, we ask whether or not the number of communities across which each k-shell is distributed is a byproduct of random interactions. To answer this question, first, for each network, we extract communities using either `Lvn` or `SBM`. Second, we compute $n_{\mathrm{C}}(k_{\mathrm{s}})$ for each k_{s}. Third, we compute the same quantity for the case in which we permute the association between the k-shell index of each node, $k_{\mathrm{s}}(i)$, and the community membership of the node, $g(i)$, uniformly at random; in fact, it is sufficient to randomly permute either $\{k_{\mathrm{s}}(1), \ldots, k_{\mathrm{s}}(N)\}$ or $\{g(1), \ldots, g(N)\}$, not both. Fourth, we calculate the number of communities to which the set of nodes with a given k_{s} value belong after the permutation, which is denoted by $n_{\mathrm{C}}^{S}(k_{\mathrm{s}})$. Fifth, using an approach similar to the calculation of the rich-club coefficient [10], we compute

$$\varphi(k_{\mathrm{s}}) = \frac{n_{\mathrm{C}}^{S}(k_{\mathrm{s}})}{n_{\mathrm{C}}(k_{\mathrm{s}})} \tag{S1}$$

for each k_{s}. A value of $\varphi(k_{\mathrm{s}})$ larger (smaller) than 1 indicates that the number of communities to which the nodes having the k_{s} value belong is smaller (larger) than in the case of the randomised association between the nodes and communities. Therefore, $\varphi(k_{\mathrm{s}})$ larger than 1 implies that the nodes with the given k-shell index, k_{s}, are concentrated into a relatively small number of communities as compared to randomised counterparts.

In Supplementary Fig. S12 we plot $\varphi(k_{\mathrm{s}})$ against k_{s} for all the data sets. We observe that, with the exception of the Spanish and British Cookpad's networks, $\varphi(k_{\mathrm{s}})$ tends to be larger than 1. This result implies that, on average, nodes of a given k-shell tend to belong to less communities than the randomised case. We stress that the permutation of either the k-shell index or the community membership sequences may return networks whose k-shell and community structure are not physically plausible. For instance, if a node i receives a k-shell index value of α upon randomisation and α is larger than k_i (*i.e.*, degree of node i), then the node cannot belong to the corresponding k-shell.

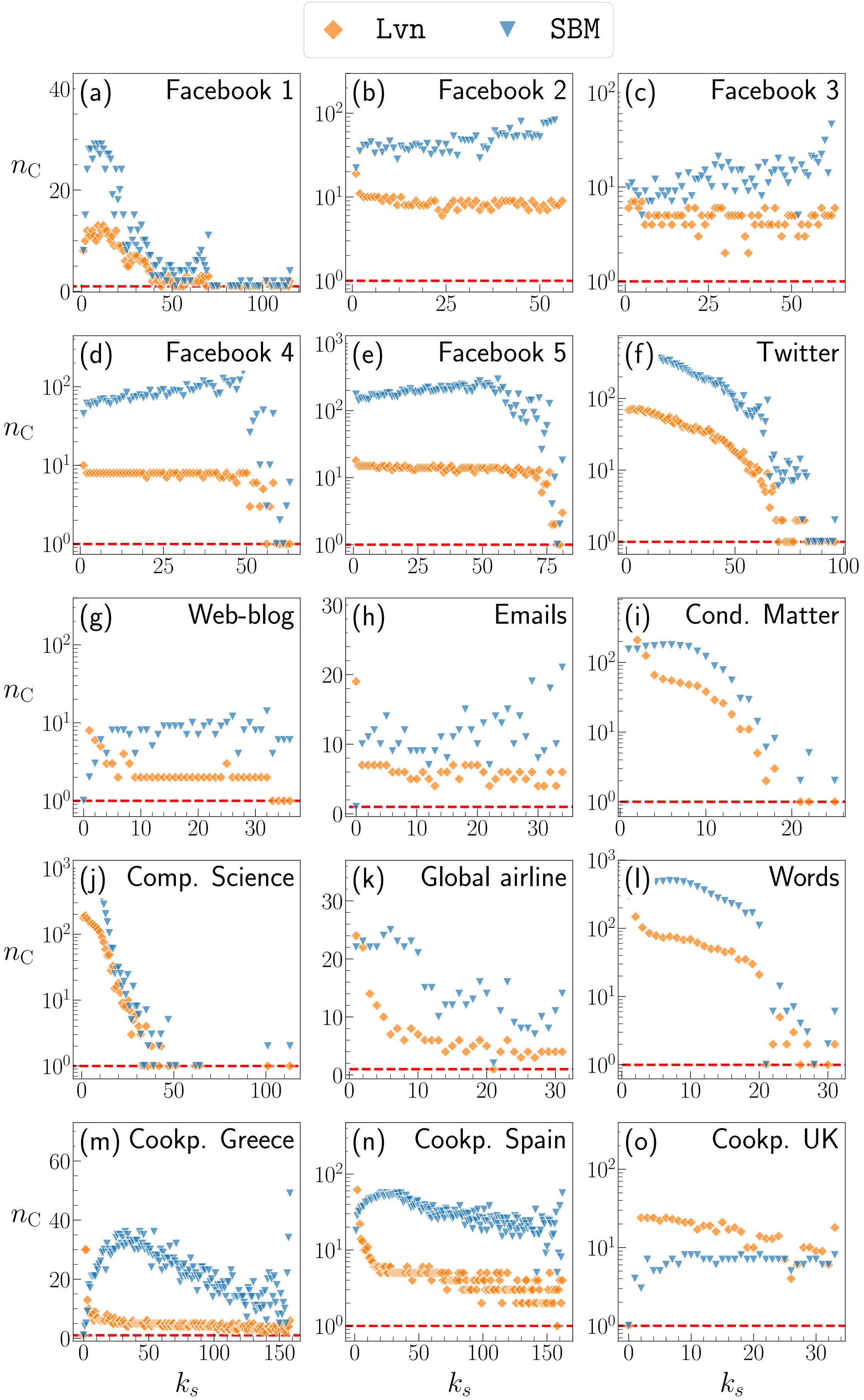

Supplementary Figure S11: Number of communities, $n_C(k_s)$, to which the nodes having k-shell index k_s belong. The horizontal line is a guide to the eyes representing $n_C(k_s) = 1$. We identified the community structure using either Lvn or SBM. Each panel accounts for a different data set.

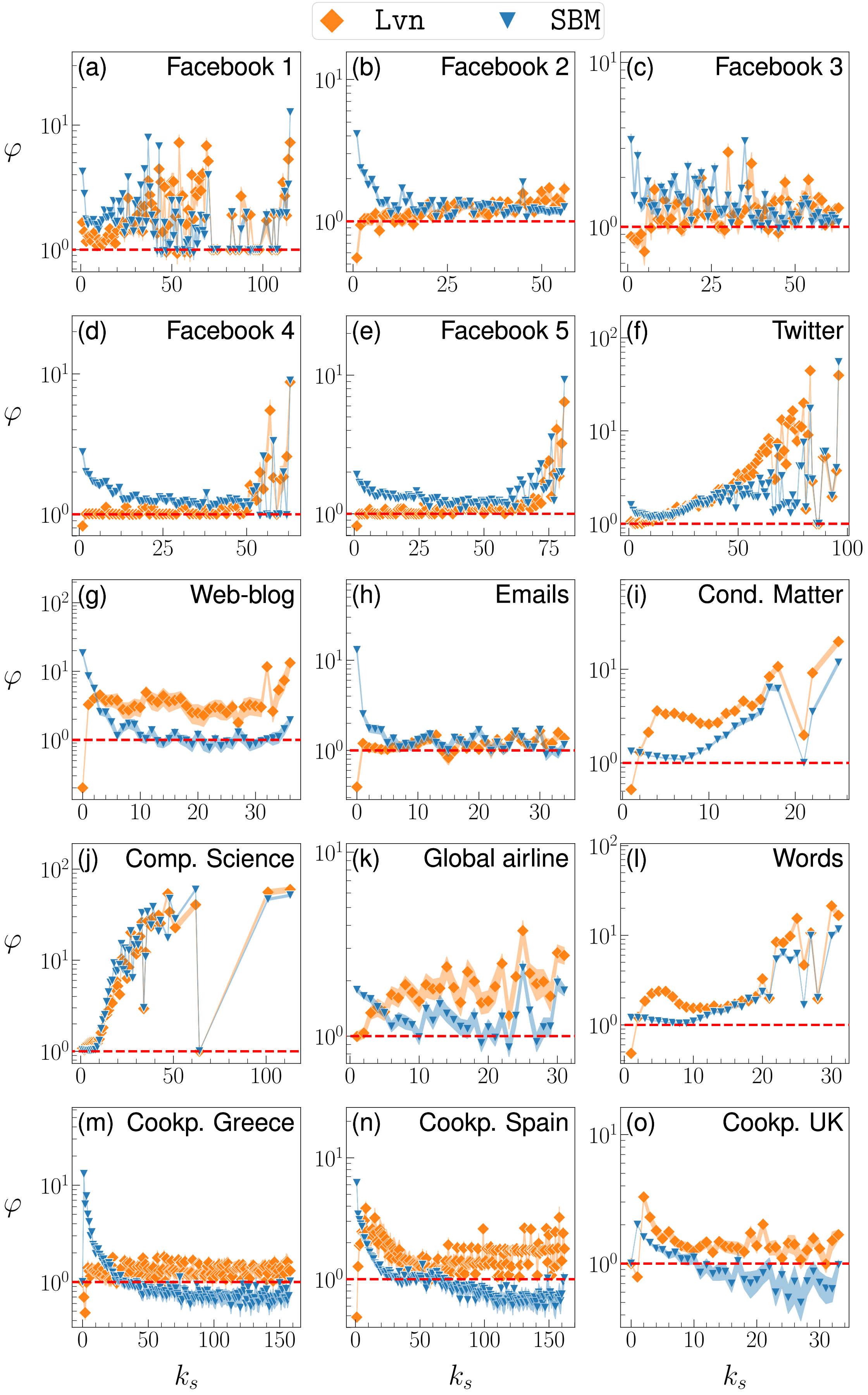

Supplementary Figure S12: Ratio, $\varphi(k_{\rm s})$, (see (S1)) plotted against the k-shell index, $k_{\rm s}$, for all the data sets. We identified the community structure using either `Lvn` or `SBM`. Each panel accounts for a different data set. Results are averaged over one hundred runs of randomisation between the association between the node's k-shell index and community label. The horizontal dashed lines represent $\varphi(k_{\rm s}) = 1$.

References

[1] Fortunato, S. & Barthélemy, M. Resolution limit in community detection. *Proceedings of the National Academy of Sciences of the United States of America* **104**, 36–41, DOI: 10.1073/pnas.0605965104 (2007).

[2] Fortunato, S. & Hric, D. Community detection in networks: A user guide. *Physics Reports* **659**, 1–44, DOI: 10.1016/j.physrep.2016.09.002 (2016).

[3] Lambiotte, R., Delvenne, J. C. & Barahona, M. Random walks, Markov processes and the multiscale modular organization of complex networks. *IEEE Transactions on Network Science and Engineering* **1**, 76–90, DOI: 10.1109/TNSE.2015.2391998 (2014).

[4] Olhede, S. C. & Wolfe, P. J. Network histograms and universality of blockmodel approximation. *Proceedings of the National Academy of Sciences of the United States of America* **111**, 14722–14727, DOI: 10.1073/pnas.1400374111 (2014).

[5] Newman, M. E. J. Equivalence between modularity optimization and maximum likelihood methods for community detection. *Phys. Rev. E* **94**, 052315, DOI: 10.1103/PhysRevE.94.052315 (2016).

[6] Young, J.-G., St-Onge, G., Desrosiers, P. & Dubé, L. J. Universality of the stochastic block model. *Phys. Rev. E* **98**, 032309, DOI: 10.1103/PhysRevE.98.032309 (2018).

[7] Lancichinetti, A., Fortunato, S. & Radicchi, F. Benchmark graphs for testing community detection algorithms. *Physical Review E* **78**, 046110, DOI: 10.1103/PhysRevE.78.046110 (2008).

[8] Amaral, L. A. N., Scala, A., Barthelemy, M. & Stanley, H. E. Classes of small-world networks. *Proceedings of the National Academy of Sciences of the United States of America* **97**, 11149–11152, DOI: 10.1073/pnas.200327197 (2000).

[9] Hagberg, A. A., Schult, D. A. & Swart, P. J. Exploring network structure, dynamics, and function using networkx. In Varoquaux, G., Vaught, T. & Millman, J. (eds.) *Proceedings of the 7th Python in Science Conference*, 11 – 15 (Pasadena, CA USA, 2008).

[10] Colizza, V., Flammini, A., Serrano, M. Á. & Vespignani, A. Detecting rich-club ordering in complex networks. *Nature Physics* **2**, 110–115, DOI: 10.1038/nphys209 (2006).
